# Rational development of fingolimod nano-embedded microparticles as nose-to-brain neuroprotective therapy for ischemic stroke

**DOI:** 10.1007/s13346-024-01721-8

**Published:** 2024-11-01

**Authors:** Xinyue Zhang, Guangpu Su, Zitong Shao, Ho Wan Chan, Si Li, Stephanie Chow, Chi Kwan Tsang, Shing Fung Chow

**Affiliations:** 1 Department of Pharmacology and Pharmacy, Li Ka Shing Faculty of Medicine, The University of Hong Kong, L2-08B, 2/F, Laboratory Block, 21 Sassoon Road, Pokfulam, Hong Kong SAR China; 2 Advanced Biomedical Instrumentation Centre, Hong Kong Science Park, Shatin, Hong Kong SAR China; 3 Clinical Neuroscience Institute, The First Affiliated Hospital of Jinan University, Guangzhou, China

**Keywords:** Nose-to-brain drug delivery, Neuroprotection, Ischemic stroke, Fingolimod, Nanoparticles, Particle engineering, Nasal powder

## Abstract

Ischemic stroke is one of the major diseases causing varying degrees of dysfunction and disability worldwide. The current management of ischemic stroke poses significant challenges due to short therapeutic windows and limited efficacy, highlighting the pressing need for novel neuroprotective treatment strategies. Previous studies have shown that fingolimod (FIN) is a promising neuroprotective drug. Here, we report the rational development of FIN nano-embedded nasal powders using full factorial design experiments, aiming to provide rapid neuroprotection after ischemic stroke. Flash nanoprecipitation was employed to produce FIN nanosuspensions with the aid of polyvinylpyrrolidone and cholesterol as stabilizers. The optimized nanosuspension (particle size = 134.0 ± 0.6 nm, PDI = 0.179 ± 0.021, physical stability = 72 ± 0 h, and encapsulation efficiency of FIN = 90.67 ± 0.08%) was subsequently spray-dried into a dry powder, which exhibited excellent redispersibility (RdI = 1.09 ± 0.04) and satisfactory drug deposition in the olfactory region using a customized 3D-printed nasal cast (45.4%) and an Alberta Idealized Nasal Inlet model (8.6%) at 15 L/min. The safety of the optimized FIN nano-embedded dry powder was confirmed in cytotoxicity studies with nasal (RPMI 2650 and Calu-3 cells) and brain related cells (SH-SY5Y and PC 12 cells), while the neuroprotective effects were demonstrated by observed behavioral improvements and reduced cerebral infarct size in a middle cerebral artery occlusion mouse stroke model. The neuroprotective effect was further evidenced by increased expression of anti-apoptotic protein BCL-2 and decreased expression of pro-apoptotic proteins CC3 and BAX in brain peri-infarct tissues. Our findings highlight the potential of nasal delivery of FIN nano-embedded dry powder as a rapid neuroprotective treatment strategy for acute ischemic stroke.

**Graphical abstract:**

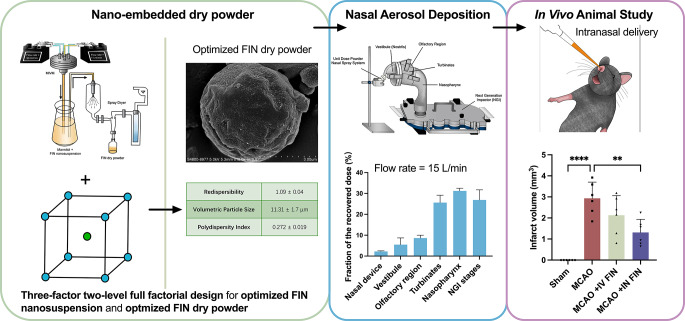

**Supplementary Information:**

The online version contains supplementary material available at 10.1007/s13346-024-01721-8.

## Introduction

Stroke is a leading cause of permanent disability worldwide, affecting approximately 15 million global citizens annually [1]. Despite ongoing improvements in stroke care and rehabilitation, many patients still suffer from different degrees of lifelong disabilities. Stroke accounts for an estimated 3–4% of total medical expenses in Western countries [1], not only affecting patients’ health and quality of life but also imposing a considerable long-term financial burden on healthcare systems. Ischemic stroke is the most prevalent form of stroke and is responsible for around 85% of stroke cases [2]. Although intravenous thrombolysis and mechanical thrombectomy are clinically available for acute ischemic stroke treatment, only a small portion of patients benefit due to a restricted therapeutic time window, resulting in a high rate of disability among stroke patients [2]. Consequently, there is a need to develop rapid and effective neuroprotective therapies for the management of acute ischemic stroke.

Upon the onset of ischemic stroke, blood supply to the neurons is interrupted immediately, resulting in substantial cell death [3]. Timely treatment has a significant impact on relieving the severity of the patient’s disease condition and their degrees of lifelong disabilities. However, it typically takes several hours for oral neuroprotective medications to reach their optimal treatment concentration in the brain. Hence, alternative drug administration methods with faster onset are required, especially for patients who are unable to take medicines orally during an acute ischemic stroke episode. Nose-to-brain drug administration has become attractive nowadays because of its minimal invasiveness, easier self-administration, higher effectiveness with less systemic side effects, and more direct route to the central nervous system [4, 5]. Previous research has indicated the drug can be delivered to the brain rapidly after intranasal administration [6]. Integrating intranasal administration with nanotechnology further enhances the potential of this delivery approach, as it prolongs drug residence time at the absorption site, increases cellular internalization, and regulates the release of encapsulated drugs [7]. Previous studies have also demonstrated that intranasal delivery of drug nanoparticles could achieve improved bioavailability [8] and high brain targetability [9] while concurrently reducing off-target concentration in the bloodstream [10]. Moreover, drug encapsulation within nanoparticles can minimize degradation risks [11] and promote rapid brain uptake [12, 13]. The above-mentioned properties are highly sought after for the intranasal administration of neuroprotective nanoparticles, especially for targeted transport to the ischemic brain.

Compared with nasal sprays, nasal powders possess unique benefits such as extended retention time and superior stability against enzymatic degradation within the nasal cavity [14, 15]. Furthermore, nasal powders can be reconstituted in an aqueous buffer as a nasal solution or suspension to offer more flexible dosing according to specific clinical needs [16]. A notable obstacle in the engineering of nanoparticle-loaded powders is the maintenance of appropriate redispersibility of dry powder back into the nanoparticle once contact with the nasal fluid to preserve the therapeutic merits of nanoparticles. Another major challenge in fabricating dry powders for optimal nose-to-brain delivery lies in particle size control, as only particles with a diameter of around 10 μm can achieve greater deposition in the olfactory region, i.e., the primary target believed to be responsible for nose-to-brain delivery [17, 18]. In the present study, spray drying was selected for converting nanosuspensions into dry powders owing to its commercial availability for simplified scale-up, as well as its ability to tailor particles for targeted intranasal delivery to the olfactory region [19, 20].

Fingolimod (FIN) is an oral drug approved for the treatment of multiple sclerosis [21]. It has also demonstrated neuroprotective effects in various animal studies and clinical trials. For instance, ischemic stroke models of mice that received FIN intravenously or intraperitoneally showed a decrease in infarct size and improved behavioral testing results [22, 23]. Moreover, stroke patients treated with standard management and oral FIN medication beyond the 4.5 h treatment window for intravenous thrombolysis (tissue plasminogen activator) displayed reduced secondary tissue injury, decreased neurological impairments, and improved post-stroke recovery compared to patients who received standard management alone [24]. Consequently, FIN is a promising neuroprotective drug with therapeutic potential for acute ischemic stroke. Curcumin (CUR), a polyphenol found in turmeric, has also shown neuroprotective effects in both hemorrhagic and ischemic stroke cases [25]. However, the delivery of FIN and CUR to the brain still requires optimization for improved clinical outcomes.

While separate studies have shown the therapeutic potential of FIN for acute ischemic stroke, no research has yet studied its neuroprotective effect through intranasal nanotherapy. This delivery strategy confers the unique advantage of rapid treatment for patients during ambulance transportation, obviating the requirement for conscious patient cooperation. Hence, the objective of this study was to develop FIN nano-embedded nasal powders for rapid neuroprotection after the onset of acute ischemic stroke. To this end, a full factorial design of experiments was conducted to examine the effects of and optimize critical formulation and processing parameters for FIN nanosuspension and its nano-embedded dry powder formulations. The optimized powder formulation was characterized by various pharmaceutical properties, such as aqueous redispersibility, particle size, nasal deposition profile, crystallinity, cytotoxicity, and stability. Lastly, the neuroprotective effects of nasally administered FIN nanoparticles were evaluated through neurological functional tests and infarct size measurements in a well-established acute ischemic stroke model.

## Materials and methods

### Materials

Fingolimod (FIN, > 99.9% purity) was purchased from Hefei Hirisun Phamatech (Hefei, China). Curcumin (CUR, > 99.5% purity) was sourced from Yung-Zip Chemicals (Taichung, Taiwan). Methanol (MeOH) and ethanol (EtOH) were obtained from VWR BDH Chemicals (VWR International S.A.S., Fontenay-sous-Bois, France). Ultra-purified water (UPW) was generated using a Barnstead NANOpure Diamond system (Thermo Fisher Scientific, Waltham, MA, USA). Polyvinylpyrrolidone (PVP K30) and cholesterol (CLT) were procured from Sigma-Aldrich (St. Louis, MO, USA), and Mannitol (Pearlitol 160 C) was purchased from Roquette (Lestrem, France). Trifluoroacetic acid (TFA), Dulbecco’s Modified Eagle Medium/Nutrient Mixture F-12 (DMEM/F12), Dulbecco’s Modified Eagle’s Medium (DMEM), Minimum Essential Medium (MEM), Kaighn’s Modification of Ham’s F-12 Medium (F-12 K medium), 0.25% (w/v) trypsin-EDTA, phosphate-buffered saline (PBS, 10×), fetal bovine serum (FBS), and antibiotic–antimycotic (100×) were purchased from Thermo Fisher Scientific (Waltham, MA, USA). Additionally, a variety of cell lines were obtained from the American Type Cultural Collection (ATCC; Manassas, VA, USA), including PC 12 cells, RPMI 2650 cells, Calu-3 cells, and SH-SY5Y cells. Nissl Staining Solution (Cat. No. C0117) was purchased from Shanghai Beyotime Biotechnology Co., Ltd (Shanghai, China). Polyvinylidene difluoride membrane was purchased from Bio-Rad (California, USA). Primary antibodies against Cleaved Caspase-3 (CC3) (Cat. No. 9664) and β-Tubulin proteins (Cat. No. #2146S) were purchased from Cell Signaling Technology (Massachusetts, USA) whose t against B-cell lymphoma 2 (BCL-2) (Cat. No. WL01556) and BCL-2 associated X (BAX) (Cat. No. WL01637) proteins were purchased from Wanlei Bio (Liaoning, China). Horseradish peroxidase-labeled goat anti-rabbit IgG(H + L) secondary antibodies (Cat. No. A0208) were purchased from Beyotime (Shanghai, China).

### High-performance liquid chromatography (HPLC)

FIN and CUR were assayed by HPLC using a C18 column (5 μm, 250 mm × 4.6 mm; Eclipse Plus; Agilent Technologies, Lexington, MA, US) coupled with a guard column (5 μm, 12.5 mm × 4.6 mm) and a photodiode array detector (Infinity 1260 LC System, Agilent Technologies, Lexington, MA, US) under a gradient mode (Table S1). The mobile phase comprised mobile phase A [0.15% TFA in UPW (v/v)] and mobile phase B [0.15% TFA in MeOH (v/v)]. A 20 µL sample was injected into the column at a flow rate of 1 mL /min. The UV detection wavelengths of FIN and CUR were set at 220 nm and 430 nm, respectively. FIN and CUR eluted at around 9.6 min and 5.3 min, respectively. The calibration curves showed excellent linearity for both FIN (*R*^2^ = 0.9997) and CUR (*R*^2^ = 0.9999).

### Preparation of FIN Nanosuspensions

**Fig. 1:**
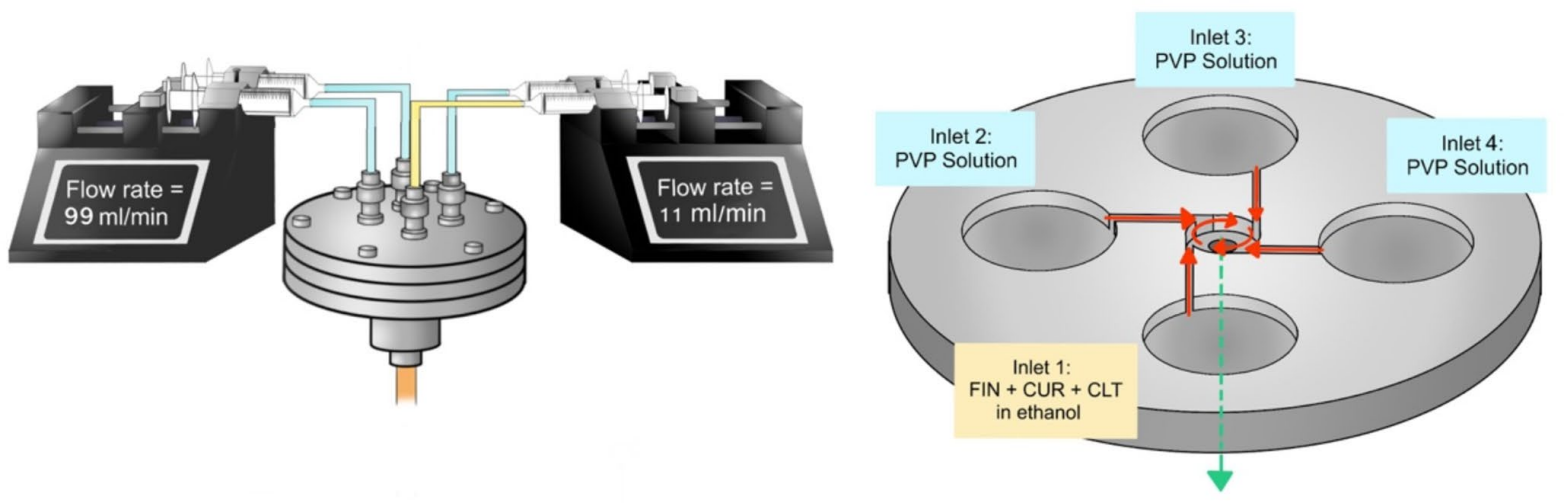
The experimental setup for the preparation of FIN nanosuspensions

The FIN nanosuspension was produced using a four-inlet multi-inlet vortex mixer (MIVM) [26], as illustrated in Fig. 1 and **Video S1**. Specifically, FIN and CUR, along with CLT, were dissolved in EtOH as an organic stream in inlet 1, while the remaining inlets (inlets 2–4) were loaded with PVP aqueous solution as antisolvent streams to create sufficient supersaturation of solutes for nanoparticle formation. The flow rates of inlets 2 and 4 were regulated by a PHD ULTRA syringe pump (Harvard Apparatus, Holliston, MA, USA) at 99 mL/min, and inlets 1 and 3 were controlled by another syringe pump (Terumo Corporation, Tokyo, Japan) at 11 mL/min. The FIN nanosuspension was collected from the outlet stream of the MIVM. The flow pattern can be characterized by calculating the Reynolds number (Re), a dimensionless number representing the ratio of inertial force to viscous force in a flowing fluid, as shown in Eq. 1. The resulting Re was fixed around 4,000 to ensure homogeneous mixing of the four inlet streams prior to nanoprecipitation [27, 28]. The production time for preparing 100 mL of nanosuspension at Re ~ 4,000 is around 0.45 min, while the characteristic mixing time of the MIVM is on the order of milliseconds [29].

1 $$\:Re=\frac{inertial\:force}{viscous\:force}=\sum\:_{i=1}^{4}\frac{{\rho\:}_{i}{v}_{i}D}{{\mu\:}_{i}}\:=\:\frac{4}{\pi\:D}\sum\:_{i=1}^{4}\frac{{\rho\:}_{i}{Q}_{i}}{{\mu\:}_{i}}\:\:$$

where *i* is the stream number, *ρ*_i_ is the fluid density (kg/m^3^), *Q*_i_ is the stream flow rate (m^3^/s), *µ*_*i*_ is the fluid viscosity (Pa´s), *v*_*i*_ is the stream velocity (m/s) and *D* is the internal diameter (1.13 × 10^− 3^ m) of the MIVM.

### Characterization of FIN Nanosuspensions

#### Particle size, size distribution and Zeta Potential

The *z*-average particle size, size distribution, and polydispersity index (PDI) of FIN nanoparticles were measured by dynamic light scattering (DLS) using a Delsa Nano C particle analyzer (Beckman Coulter, Brea, CA, USA). The viscosity and refractive index of the medium were assumed to be the same as those of pure water (0.89 mPa·s and 1.331 at 25 °C). The zeta potential of FIN nanoparticles was determined using the same particle analyzer mentioned above. The measured electrophoretic mobility was converted into zeta-potential using the Smoluchowski relationship [30].

#### Physical Stability

The physical stability of FIN nanosuspension was monitored by measuring the change in particle size over time [31]. The test was terminated when either a > 20% change in particle size occurred or visible precipitation was observed in the nanosuspension.

#### Encapsulation Efficiency (EE) and drug loading (DL) of the Nanosuspension

The determination of EE and DL was performed using an established protocol [32]. Briefly, 15 mL of FIN nanosuspension was transferred into an Amicon^®^ Ultra 30 kDa centrifugal filter device (Sigma Aldrich, St Louis, MO, USA) and centrifuged at 4000 x *g* for 40 min. The filtrate, containing free FIN and CUR, was collected for HPLC analysis while the concentrated sample was freeze-dried using a Freezone 6 L Benchtop Freeze Dry System with Stoppering Tray Dryer (Labconco Corporation, Kansas City, MO, US). The freeze-dried product was weighed and dissolved in a mixture of UPW and MeOH with 0.15% TFA [23:77 (*v/v*)] for HPLC analysis of the drug content in the nanoparticles. The EE and DL were then calculated according to Eqs. 2 and 3, respectively.

2 $$\:\text{E}\text{E}\:\left(\%\right)=\frac{total\:amount\:of\:drug-amount\:of\:free\:drug}{total\:amount\:of\:drug}\times\:100\%\:$$

3 $$\:D\text{L}\:\left(\%\right)=\frac{total\:amount\:of\:drug\:in\:nanoparticles}{total\:amount\:of\:nanoparticles}\times\:100\%\:$$

#### Transmission Electron Microscopy (TEM)

A single drop of freshly produced FIN nanosuspension was dripped on a carbon film TEM grid that had been discharged using the PELCO easiGlow™ Glow Discharge Cleaning System (Redding, CA, US), followed by staining with 2% (*v/v*) uranyl acetate for 1 min. Then, the Tecnai™ G2 20 S-TWIN Transmission Electron Microscope (FEI, Hillsboro, OR, US) was employed to image the nanoparticles on the air-dried grid at ×19,500 magnification.

### Design of experiment (DoE)-guided optimization of FIN Nanosuspension

A 2-level, 3-factor full factorial design was employed to examine the main effects and interactions of selected formulation parameters on the critical quality attributes (CQAs) of the FIN nanosuspension (Table S2). The processing parameters were the initial concentration of FIN (mg/mL) (A), the mass ratio between CLT and FIN (w/w) (B), and the concentration of PVP solution [% (w/v)] (C), while the responses included particle size (nm) (Y_1_), PDI (Y_2_), physical stability (hours) (Y_3_), and EE of FIN (%) (Y_4_). The initial concentration of CUR was fixed at 2.5 mg/mL. The levels of each variable were set as + 1, 0, and − 1, and a total of 9 experimental runs (2^3^ + 1 runs as the center point) were performed.

The optimal processing parameters of FIN nanosuspension were identified using the desirability function approach based on the regression models constructed by Design-Expert 13 for the responses [33]. Each response was associated with a partial desirability function (*d*), where a fully desired response was assigned a value of 1 and an unfavorable response was assigned a value of 0. The overall desirability value (*D*) was determined using the geometric mean of the partial desirability functions.

### Preparation of FIN Nano-embedded Microparticle Dry Powder formulations

FIN nano-embedded microparticle dry powder formulations were produced using spray drying. Specifically, mannitol solution was mixed with freshly prepared optimized FIN nanosuspension, and this mixture was fed into a Büchi spray dryer (Mini Spray Dryer B-290 coupled with Dehumidifier B-296, Flawil, Switzerland) with nitrogen as the drying gas [32]. The aspiration rate and inlet temperature were fixed at 100% (38 m^3^/h) and 110 ℃, while the atomization flow rate and feed rate were varied in each run according to the experimental design. The processing time for fabricating spray-dried powder depended on the total volumes of nanosuspension and mannitol solution, as well as the feed rate of the solution during the spray drying process. In this work, when the volumes of both the nanosuspension and mannitol solution were 60 mL, the processing time was approximately 80 min under a feed rate of 1.5 mL/min. The resulting product was collected in a Falcon 50 mL conical tube and transferred to a desiccator upon spray drying.

### Characterization of FIN Nano-embedded Microparticle Dry Powder Formulation

#### Aqueous redispersibility

The redispersibility test was performed by reconstituting the spray-dried powder in UPW at room temperature. Briefly, 15 mg of dry powder was transferred into 10 mL of UPW, and the resulting suspension was stirred at 75 rpm for 10 min. After 3 min, the particle size was measured as described in Section Particle Size, Size Distribution and Zeta Potential. The redispersibility index (RdI) was denoted as S_f_/S_i_, where S_i_ and S_f_ represent the particle size of FIN nanoparticles before and after spray drying, respectively [34]. A RdI value of 1 indicates that the particle size remains unchanged during the spray drying process.

#### Particle size distribution by laser diffraction

A HELOS/KR laser diffractometer (Sympatec, Germany) was used to determine the volumetric size distribution of the dry powder, as previously reported with minor modifications [35]. Briefly, a Unidose powder nasal spray system (Aptar Pharma, France) filled with 5.0 ± 0.5 mg dry powder was connected to the laser diffractometer (30^◦^ angle) using an adaptor. The dry powder was then dispersed at a flow rate of 15 L/min. The spherical volume diameters at 10% (D_10_), 50% (D_50_), and 90% (D_90_) cumulative volumes were recorded. The span of the dry powder was expressed as (D_90_ − D_10_)/ D_50_. Each sample was measured three times.

#### In Vitro Evaluation of Nasal Aerosol Depositions

##### 3D-printed nasal cast model

**Fig. 2:**
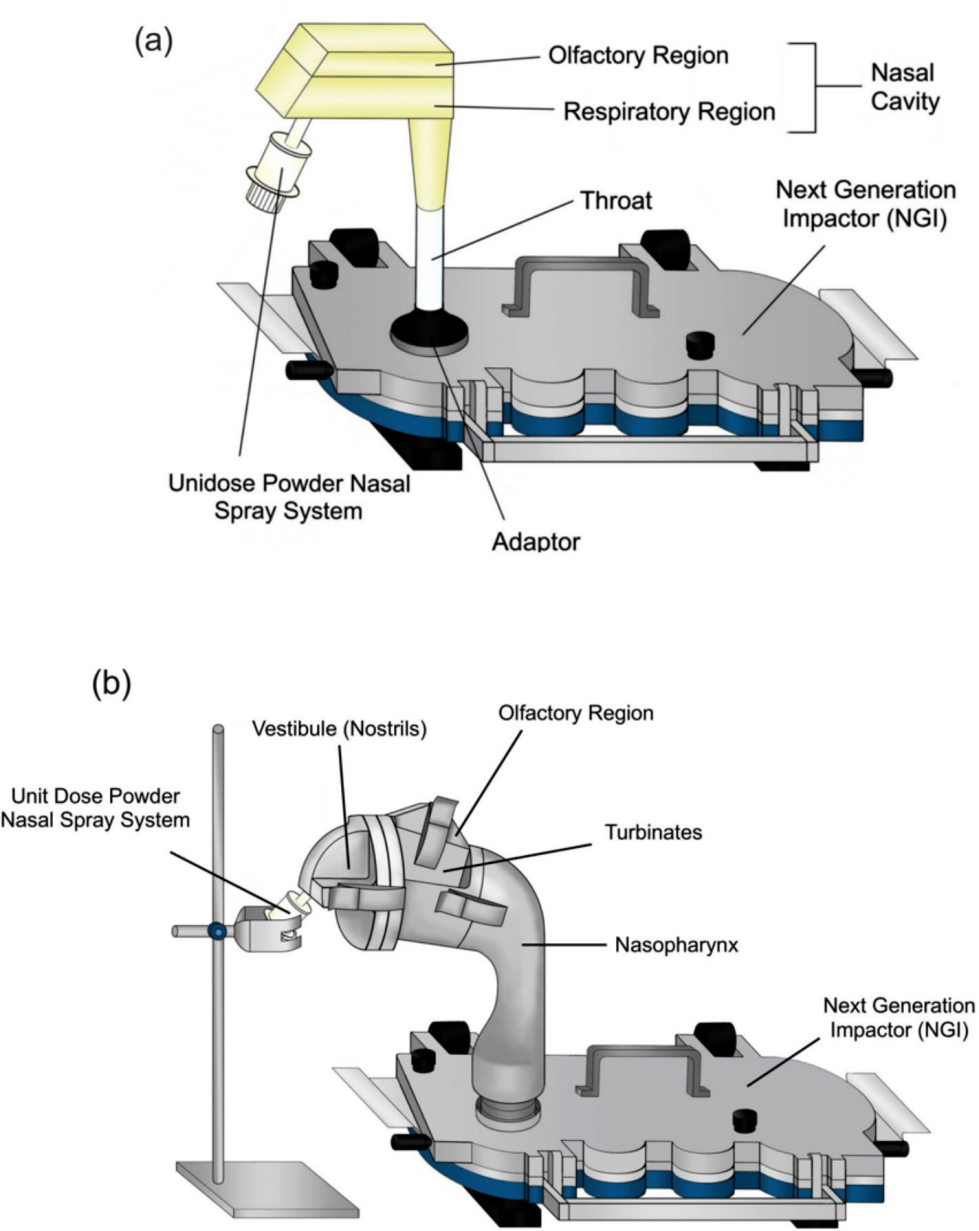
Schematic presentation of (**a**) the 3D printed nasal cast; (**b**) the AINI coupled with the NGI for the aerosol deposition assessment

As depicted in Fig. 2a, a Next Generation Impactor (NGI) (Copley, Nottingham, UK) coupled with a customized 3D-printed nasal cast model was employed to assess the in vitro aerosol performance of the optimized FIN nano-embedded dry powder formulation [36]. The 3D-printed nasal cast can be dismantled into two parts: the olfactory region and the respiratory region. A thin layer of silicon grease (Slipicone; LPS Laboratories, Tucker, GA, USA) was sprayed on all stages of the NGI to minimize particle bouncing. An unidose powder nasal spray device (Aptar Pharma, France) was used to disperse 15.0 ± 1.0 mg of optimized FIN nano-embedded dry powder into the nasal cavity under inspiratory flow rates of 0, 7.5, and 15 L/min. The insertion depth and the insertion angle of the nasal device into the nostril were 5 mm and 60^◦^ from the horizontal plane, respectively. After dispersion, the powder in the 3D-printed nasal cast model and NGI stages was rinsed using the HPLC mobile phase (UPW and MeOH with 0.15% TFA [23:77 (v/v)]). The resulting solution was filtered through a 0.45-µm membrane for FIN assay. The deposition experiment was repeated thrice.

The recovered dose was defined as the total mass of FIN recovered in the 3D-printed nasal cast model and the NGI stages, and all fractions were calculated with respect to the recovered dose. The nasal device fraction was calculated with the powder mass remaining in the nasal spray device after dispersion, while the throat fraction was calculated using the powder mass deposited in the throat and adaptor regions connecting the NGI and nasal cast. The NGI stages fraction was calculated based on the powder mass deposited in Stages 1–7 and the MOC of the NGI.

##### Alberta Idealised Nasal Inlet (AINI) Model

An Alberta Idealised Nasal Inlet (AINI) (Copley, Nottingham, UK) coupled to the NGI was also utilized to examine in vitro nasal aerosol deposition (Fig. 2b) [4]. This model was created based on a set of realistic nasal anatomies and composed of four detachable components, i.e., olfactory region, vestibule, turbinates, and nasopharynx [37–40]. The procedures for coating the NGI stages, filling the nasal spray device, inserting the nasal spray device, FIN assay, and calculations of the recovered dose and nasal device fraction were the same as those described in Section 3D-printed Nasal Cast Model.

#### Scanning Electron Microscopy

A Hitachi S-4800 FEG field emission scanning electron microscope (Hitachi, Tokyo, Japan) was utilized to characterize the particle morphology of the spray-dried powder at 5.0 kV. The powder sample was gently dispersed on carbon tape mounted on a SEM stub and subsequently coated by an ~ 11 nm gold-palladium alloy using a sputter coater for 90 s to prevent charging interferences during the imaging.

#### Powder X-Ray diffractometry (PXRD)

A Rigaku SmartLab 9 kW diffractometer with a copper rotating anode (K alpha1 1.54059 Å, K alpha2 1.54441 Å) rated at 160 mA/ 45 kV was used to collect the PXRD patterns of the samples. A K beta nickel filter was used to filter the diffraction signals. Each sample was scanned within a 2θ range of 3° to 40°, with a step width of 0.02° and a scanning speed of 5.0° per minute.

#### Fourier-Transform Infrared Spectroscopy (FTIR)

A Spectrum Two FTIR spectrometer (Perkin Elmer, Waltham, MA, USA) was used to generate FT-IR spectra in KBr diffuse reflectance mode. The scan was performed in the range of 4,000 cm^− 1^ to 1,000 cm^− 1^ at intervals of 0.5 cm^− 1^. A total of 32 scans were performed at a resolution of 4 cm^− 1^ for each sample.

#### Differential scanning calorimetry (DSC)

The thermal characteristics of the samples were measured using a DSC 250 differential scanning calorimeter (TA Instruments, New Castle, DE, USA). Prior to the measurement, pure indium was used for calibration. Each sample (3 ± 0.5 mg) was encased in a Tzero hermetic pan and heated from 50 °C to 250 °C at a ramp rate of 10 °C/min under an N_2_ flow rate of 20 mL/min.

#### Thermogravimetric analysis (TGA)

The residual solvent and moisture content (*M*) of the spray-dried powder was determined using a TGA550 thermogravimetric analyzer (TA Instruments, Newcastle, DE, USA) according to Eq. (5). Approximately 3 mg of sample was loaded in a platinum pan and heated from 25 °C to 200 °C at a scanning rate of 10 °C/min under a N_2_ flow rate of 20 mL/min.

4 $$\:M\:\left(\%\right)=\frac{{m}_{0}-{m}_{1}}{{m}_{0}}\times\:100\:$$

where m_0_ and m_1_ represent the weight of the measured sample before and after the experiment, respectively.

#### Encapsulation Efficiency (EE) and drug content of powders

To determine the encapsulation efficiency of powder, the powder should be first converted back to the reconstituted nanosuspension. Hence, 20 mg of spray-dried dry powder was dispersed into 10 mL of UPW under a stirring rate of 75 rpm for 10 min to gain reconstituted nanosuspension. The reconstituted nanosuspension was subsequently transferred into the filter device (Amicon^®^ Ultra-15, Sigma Aldrich, St Louis, MO, USA) and centrifuged at 4000 x g for 40 min. The analytical procedure was the same as mentioned in Section Encapsulation Efficiency (EE) and Drug Loading (DL) of the Nanosuspension. The purpose of assessing the EE of the dry powder is to verify whether any drug loss occurred during the spray drying process. Regarding the drug content of FIN and CUR in the spray-dried dry powder, 10 mg of sample was accurately weighed and transferred into the tube and dissolved in a 2 mL mixture of UPW and MEOH with 0.15% TFA [23:77 (*v/v*)] for HPLC assay of FIN and CUR in the sample [32, 41].

#### Stability studies

The samples were stored in screw-capped glass tubes at 4 °C, room temperature, and 40 °C under 30% relative humidity for 2 months. The chemical stability of the sample was monitored by conducting HPLC assays of FIN and CUR, while the physical stability of the sample was checked by the DSC, TGA, and PXRD analysis.

#### In Vitro Drug Release

The drug release profiles of the FIN dry powder were obtained using a reported protocol with modifications [42]. A total of 50 ± 0.5 mg of FIN dry powder or its physically mixed counterpart, which was based on the components of the spray-dried FIN powder manually mixed using a mortar and pestle to match the composition of the spray-dried FIN powder, was dispersed into 20 mL of simulated nasal fluid. The resulting solution was stirred at 75 rpm for 3 h at 34 ± 0.1 °C. The compositions of simulated nasal fluid included NaCl (7.45 g/L), KCl (1.29 g/L), CaCl_2_ × 2 H_2_O (0.32 g/L), and double-distilled water, with the pH adjusted to 6.4 [43]. A 0.5 mL aliquot of the solution was withdrawn at designated time points (15, 30, 60, 120, and 180 min) and transferred into an Amicon^®^ Ultra-0.5 centrifugal filter unit. Medium replacement was carried out upon each sample collection. The FIN content in the filtrate was assayed by HPLC. The experimental procedure was repeated with a change of the medium to a mixture of simulated nasal fluid and ethanol [80:20 (v/v)].

### DoE-guided optimization of FIN Nano-embedded dry powder formulation

The experimental design, optimization, and model validation of the fabrication of FIN dry powder formulation were the same as the FIN nanosuspension production described in Section Design of Experiment (DoE)-guided Optimization of FIN Nanosuspension. Herein the formulation and processing parameters in the DoE (Table S3) included the ratio between mannitol and nanoparticle (w/w) (A), the atomization gas flow rate (L/h) (B), and the feed rate of solution during the spray drying process (mL/min) (C), while the responses included redispersibility of the dry powder (Z_1_), PDI of the reconstituted nanosuspension (Z_2_), and volumetric particle size of dry powder (µm) (Z_3_). Again, the optimal processing parameters of FIN co-loaded dry powder were identified by the desirability function approach based on the regression models constructed by Design-Expert 13 for the responses.

### In Vitro Cellular Study

#### Cell Culture

SH-SY5Y cells were cultured in DMEM culture medium, and PC 12 cells were cultured in F-12 K nutrient mixture medium containing L-glutamine. RPMI 2650 cells were cultured in MEM, while Calu-3 cells were cultured in DMEM/F12 culture medium. These cell culture mediums were supplemented with 10% (v/v) FBS and 1% (v/v) antibiotic-antimycotic. The cells were cultured in a humidified incubator at 37 °C and 5% CO_2_ and were passaged in different ratios when the cell density reached 80% confluence. 0.25% trypsin-EDTA was used to detach the cells from the T75 flasks.

#### In Vitro Cellular viability assay

Cells were seeded in 96-well plates at a density of 2 × 10^4^ cells/well (for RPMI 2650 and PC-12), 0.5 × 10^5^ cells/well (for SH-SY5Y), or 1 × 10^5^ cells/well (for Calu-3) and cultured in a humidified incubator at 37 °C and 5% CO_2_ overnight. On day 2, the cells were treated with reconstituted optimized FIN nano-embedded dry powder, raw FIN, and a physical mixture of FIN powder formulation components at FIN concentrations ranging from 2.5 to 1000 nM for 24 h. The reconstituted optimized FIN nano-embedded dry powder was prepared by redispersing optimized FIN nano-embedded dry powder in the cell culture medium. Raw FIN and their physical mixture were dissolved in DMSO and further diluted by the cell culture medium to a desired concentration. Considering the cytotoxicity of DMSO, cells were also treated with raw DMSO, which was the same as the DMSO concentration in the treatment groups as the control group. After 24 h, the cells were incubated with 100 µL of cell culture medium with 10% CCK-8 solution for 3 h. The spectrophotometric absorbance of the sample was measured at 450 nm using a microplate spectrophotometer (MultiSkan Go, Thermo Fisher Scientific, Waltham, MA, USA). The cell viability was calculated as the percentage of the absorbance from the treatment groups divided by the absorbance from the control group. All measurements were performed in triplicate.

### In vivo animal study

#### Animals and Ethics statements

All animal studies followed the ethical policies and guidelines recommended by ARRIVE (Animal Research: Reporting of In Vivo Experiments) and National Research Council’s Guide for the Care and Use of Laboratory Animals and were approved by the Ethics Committee of the Animal Experimentation of Jinan University (Approval No.: 20230901-0012). Male C57BL/6 mice (6–8 weeks old) were obtained from Zhuhai Baishitong Biotechnology Co., Ltd and were used for the ischemic stroke models. All mice were kept in a daily cycle of 12 h of light and 12 h of darkness with humidity and temperature controls. Water and food were freely available to the mice at all times in their cages.

#### Development of the ischemic stroke model

Electrocoagulation was used to create the middle cerebral artery occlusion (MCAO) model based on a reported protocol on day 1 [44]. Mice were anaesthetized using 1% (w/v) pentobarbital sodium at a dose of 50 mg/kg by intraperitoneal injection. Subsequently, a skin incision was made between the ear and right eye to expose the skull and temporalis muscle. The middle cerebral artery was exposed by penetrating the skull with a microdrill. Finally, a small vessel electrocoagulator was utilized to cauterize the distal middle cerebral artery. Following the restoration of the temporalis, a surgical suture was used to close the wound in the head skin.

#### Animal grouping

A total of 32 mice were randomly divided into 4 groups (*n* = 8 per group): (1) sham-operated group (Sham group); (2) MCAO group receiving 0.9% saline solution intranasally (IN) (MCAO group); (3) MCAO group treated with the optimized FIN nano-embedded dry powder (1 mg/kg of FIN) reconstituted in 0.9% saline solution via intravenous (IV) injection (MCAO + IV FIN group); and (4) MCAO group treated with the optimized FIN nano-embedded dry powder (1 mg/kg of FIN) reconstituted in 0.9% saline solution (~ 15 µL) intranasally (IN) (MCAO + IN FIN group). The experimental procedure for the Sham group was designed to closely parallel that of the other study groups as described in Section Development of the Ischemic Stroke Model, with the exception of the cauterization of distal middle cerebral artery. The initial dose was administered 30 min following the ischemic stroke surgery, and the second dose was given 24 h post-surgery. On the third day, the mice were sacrificed, and brain tissues were collected for western blot analysis.

#### Behavioural assessments and neurological function evaluation

The neurological recovery of mice was assessed using the adhesive removal test balance beam test, and rotarod test on days 2 and day 3 [45–47]. Baseline behavioural assessment was conducted one day prior to the stroke surgery. In the adhesive removal test, mice were placed in a container, and the time for the mice to remove the adhesive tapes from the bilateral paws was recorded. For the balance beam test, the time taken for the mice to walk across a beam (1.20 m in length, 1.0 cm in width, and 0.7 m in height) was recorded after training. In the rotarod test, mice were trained at constant speed three times before the surgery and the retention time for mice to remain on the rotarod with accelerated speed (starting at 4 rpm and increasing by 46 rpm per 60 s until the 50 rpm) was assessed. Furthermore, the Longa score was used to assess the neurological deficits of mice on day 3, especially the motor function of mouse [48, 49]. The weight of mice was monitored daily.

#### Histological Assessment

The histological assessments were conducted on day 2 after the behavioural assessments and neurological function evaluation as previously described [50]. Briefly, the mice were anesthetized (1% pentobarbital sodium, 50 mg/kg) and were perfused with 0.9% sodium chloride transcardially. After fixing with 4% paraformaldehyde, the brain was removed rapidly and dipped into the wax, followed by embedding and sectioning in 4 μm thickness. The brain slice was observed at the posterior and anterior levels based on Paxinos and Franklin’s mouse brain in stereotaxial coordinates. The brain slices were defatted by xylene and dehydrated by alcohol. For Nissl staining, the brain slices were immersed in 0.1% cresol violet for 30 min at room temperature. The brain slices were dehydrated after rinsing by UPW, followed by transparentizing and sealing with neutral gum. The TissueFAXS PLUS system from TissueGnostics was employed to scan slides automatically.

#### Magnetic resonance imaging (MRI)

The MRI was conducted on day 2 using a reported protocol with modification [51]. The 9.4 T small animal MRI scanner (Bruker PharmaScan) was used to obtain MRI images of the mice 24 h after the stroke surgery. The parameters of the T2-weighted imaging (T2WI) imaging were set as a field of view (FOV) = 20 × 20 mm, 17 axial slices with a slice thickness of 1 mm, a matrix of 256 × 256 and 2D fast-spin echo sequence (3500/33 ms of repetition time/echo time, 2 average). After anesthetization, the respiratory rate and the temperature of mice were monitored during the imaging process. The machine was placed over the brains of mice, followed by the scanning by T2WI imaging and quantitively analysis using a 3D slicer software. Based on the T2WI images, the 3D slicer was used to reconstruct the 3D images. The infarct and non-infarct zones were separated using threshold modification.

#### Western blot analysis

The western blot analysis was conducted on day 3 according to a reported protocol with modifications [45]. The RIPA buffer was used for lysing and extracting proteins from the mouse brain. Protein samples (5–30 µg/lane) were separated by 10% sodium dodecyl sulfate-polyacrylamide gel electrophoresis (SDS-PAGE) and subsequently transferred to a polyvinylidene difluoride membrane (Bio-Rad). The membrane was blocked using TBST buffer containing 5% milk for one hour, followed by overnight incubation with primary antibodies against CC3, BCL-2, BAX, and β-Tubulin (as internal control) proteins at 4℃ in 5% milk-TBST. The blot was then washed and incubated with secondary antibodies in TBST buffer containing 5% milk for one hour at room temperature. The ChemiDoc Touch imaging system (Bio-Rad) was used to detect the protein bands, while Image Lab software and Tanon 2500 Gel Imaging System were employed for data analysis. Protein bands were analyzed and quantified using Quantity One (Bio-Rad) or ImageJ with normalization provided by the respective loading controls in the same samples.

### Statistical analysis

Data were shown as mean ± standard deviation (S.D.). The optimal processing parameters of FIN nanosuspension and dry powder were obtained by Design-Expert (Version 21.3.1; Stat-Ease, Inc., Minneapolis, USA) using least-square regression and nested ANOVA of the responses. Trios (Version 5.1.1; TA Instrument, New Castle, DE, USA) was used for the analysis of DSC data. A *p*-value < 0.05 was considered statistically significant.

## Results and discussion

### Optimization of FIN Nanosuspensions

The selection of formulation parameters and the corresponding design space (Table S2) in the factorial design for fabricating FIN nanosuspensions were guided by preliminary data and technical considerations for nose-to-brain drug delivery. Without the aid of stabilizers, the resulting nanosuspensions exhibited visible precipitation within 5 min right after production, while the incorporation of PVP and CLT into the formulations significantly enhanced the physical stability of nanosuspensions (Table S4). Regarding the response specifications, as the diameter of olfactory axon in humans falls within a typical range of 100–700 nm, and previous research has demonstrated that nanoparticles with a size of less than 200 nm can be transported effectively to the brain via transcellular neuronal absorption, the target size for FIN nanoparticles was set in the range of 0–200 nm [52, 53]. In addition, considering that the nanosuspensions would be spray-dried into dry powders, they should remain colloidally stable before the drying process. Therefore, the physical stability was set as over 24 h, allowing sufficient transit time in real manufacturing practice. To unleash the therapeutic potential of the formulations, targets for the EE of FIN and PDI of the nanoparticles were set in line with the literature standards at 90% and < 0.3, respectively [32, 54].

Although single-factor experiments are frequently used for optimizing drug formulations and manufacturing processes, it is relatively time-consuming and incapable of identifying potential interaction effects of factors on responses [55, 56]. Consequently, a full factorial DoE was employed in this study. Table 1 presents the physicochemical properties of FIN nanosuspensions obtained from the DoE. The particle size of the nanosuspensions ranged from 107.4 ± 5.4 nm to 187.2 ± 2.4 nm, which were all within the target range. The PDI of these formulations, except for NF2, satisfied the pre-set requirement of < 0.3. Notably, there was a discrepancy in the stability among these formulations. For example, NF2 and NF5 showed precipitation within 30 min, whereas NF3 remained stable for at least 72 h. The EE of FIN among these formulations varied from 60.51 ± 1.84% to 95.73 ± 0.04%, while the EE of CUR consistently surpassed 99.99%. This indicates that almost all CUR could be encapsulated into the nanoparticles. The coded regression equations for each response model of the FIN nanosuspensions are shown in Table S5. These models were tested by two-way nested ANOVA and displayed statistical significance (*p* < 0.0001) for all responses, suggesting that the factors and their interactions were significantly correlated with the responses. They were also considered reliable with predicted *R*² values close to or exceeding 0.85, which agreed with the adjusted *R*² values. All regression models were therefore used to probe the relative effects of factors and their interactions on the responses.

**Table 1:** Physicochemical properties of FIN nanosuspensions prepared based on the 3-factor 2-level factorial design (*n* = 3)

Nanosuspension Formulation (NF)	Factors with levels			Responses and results			
	A	B	C	Particle Size (nm) (Y_1_)	PDI (Y_2_)	Physical Stability (hours) (Y_3_)	EE of FIN (%) (Y_4_)
NF1	5	0.5	0.25	136.6 ± 1.3	0.238 ± 0.008	0.7 ± 1.2	88.87 ± 1.25
NF2	5	0.5	1	107.4 ± 5.4	0.381 ± 0.072	< 0.5 ± 0	60.51 ± 1.84
NF3	5	1.5	0.25	134.0 ± 0.6	0.179 ± 0.021	72.0 ± 0	90.67 ± 0.08
NF4	5	1.5	1	142.8 ± 2.2	0.251 ± 0.044	40.0 ± 13.9	68.44 ± 0.56
NF5	10	0.5	0.25	155.2 ± 1.0	0.171 ± 0.019	< 0.5 ± 0	95.03 ± 0.27
NF6	10	0.5	1	166.4 ± 5.5	0.208 ± 0.025	1.3 ± 1.2	81.44 ± 0.41
NF7	10	1.5	0.25	151.8 ± 2.4	0.181 ± 0.016	6.0 ± 0	95.73 ± 0.04
NF8	10	1.5	1	187.2 ± 2.4	0.186 ± 0.006	24.0 ± 0	85.04 ± 0.45
NF9	7.5	1	0.625	154.8 ± 4.7	0.205 ± 0.013	6.0 ± 0	84.93 ± 1.73

A: Initial FIN concentration (mg/mL); B: CLT: FIN (w/w); C: Concentration of PVP solution (% w/v)

**Fig. 3:**
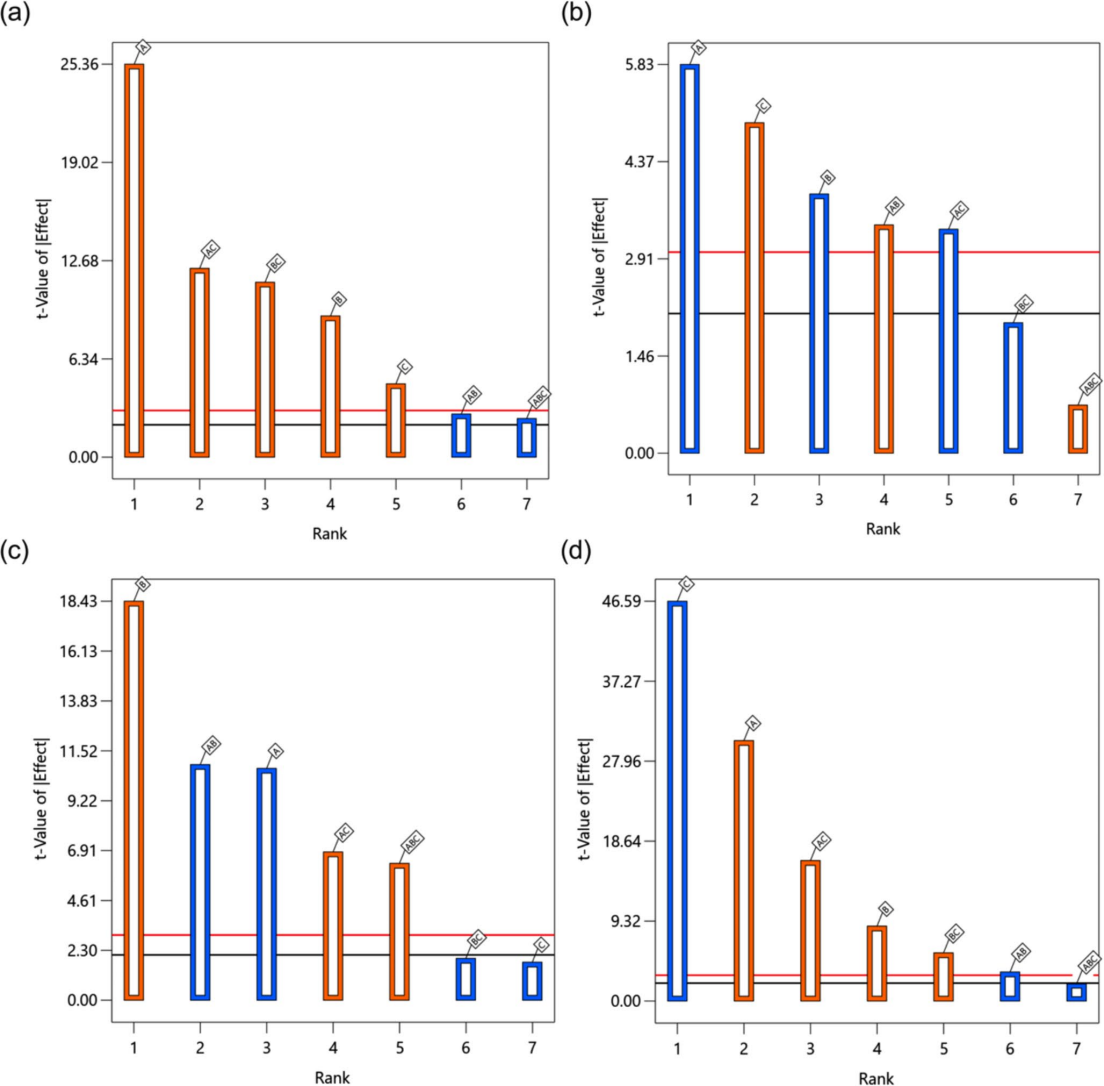
Pareto charts of standardized effects of factors and their interactions on (**a**) particle size of the FIN nanosuspension, (**b**) PDI of the FIN nanosuspension, (**c**) stability of the FIN nanosuspension and (**d**) EE of FIN nanosuspension. Factors and interactions that cross the horizontal black line show statistically significant effects on the responses. A blue bar stands for negative effects on the responses, while orange bar stands for positive effects on the responses

The Pareto charts in Fig. 3 show the standardized effects of factors and their interactions on the responses. All studied factors and their interactions exerted a significant effect on the particle size of FIN nanosuspensions (Y_1_). Specifically, increasing the initial FIN concentration (A), CLT: FIN ratio (B), the concentration of PVP solution (C), and their interaction terms AC and BC tended to produce larger particles. The observations in the main effects could be partly explained by the nucleation and growth theories. Upon bulk mixing of the aqueous and organic streams in the MIVM, an upsurge in the supersaturation of hydrophobic solutes (FIN, CUR, and CLT) would concurrently increase their nucleation and particle growth rates, resulting in a contrasting effect on the particle size. Quenching further particle growth requires adequate deposition of the water-soluble stabilizer (PVP) on nanoparticles (i.e., nuclei with dimensions beyond the critical size) via molecular diffusion, which is a time-dependent process [57]. This explains why a sole rise in factor A or B led to an increase in particle size, as the resulting nanoparticles were not instantly stabilized by PVP, and their effects on particle growth dominated that of nucleation. It is worth noting that factor C could influence particle size in both negative and positive ways. While PVP could inhibit nanoparticle growth and aggregation by surface capping, it also acted as a solubilizing agent to alter the supersaturation levels of FIN and CLT in the systems and, consequently, their nucleation kinetics. The latter effect was more apparent in the current design space. The dual roles of PVP also caused factor C to have a lower *t*-value in two-way nested ANOVA and coefficient in the regression equation compared to the other main factors. Surprisingly, the interaction terms AB and ABC, albeit marginally statistically significant, were negatively correlated with particle size. We speculate that when both factors A and B increased, the precipitated hydrophobic CLT was able to shorten the diffusion path and time of PVP from the bulk solution to the nuclei, thereby yielding smaller nanoparticles. However, the molecular interplays in nanoparticle formation and stabilization for a multi-component system are complex, and further research (e.g. using molecular dynamics simulation) is warranted to delineate their exact mechanisms.

Pertaining to the PDI (Y_2_), physical stability (Y_3_), and EE of FIN (Y_4_) responses, the factor-response relationships elucidated from the models generally aligned with the findings derived from the response model of particle size (Y_1_) and our expectations. As mentioned earlier, augmenting factor A or B would elevate the supersaturation level of the hydrophobic solutes, thereby facilitating the entrapment of more FIN molecules within the nanoparticle core during the FNP process. The resulting larger nanoparticles also possessed lower surface energy, which served to minimize particle aggregation and produce a more monodisperse nanosuspension. It is not surprising that factor C and its related 2-factor interactions (i.e., AC and BC) displayed erratic effects on the PDI, EE, and stability since PVP serves multiple functions as a crystallization inhibitor, solubilizer, and stabilizer in the nanosuspension formulation as aforementioned. Briefly, factor C had a positive, negative, and insignificant effect on PDI, EE, and stability, respectively, but its interaction factors AC and BC showed divergent trends (AC: negative for PDI, positive for EE and stability; BC: insignificant for PDI and stability, positive for EE). It should be noted that these regression models are of good predictive ability within our design space but their applicability to other systems may be limited, particularly if the design spaces of those systems differ significantly from the present study.

The regression models were simplified by eliminating non-statistically significant terms (Table 2), and the desirability function was applied to optimize the FIN nanosuspension, and the highest desirability value (0.951) was obtained under the condition of A = 5 (mg/mL), B = 1.5 (*w/w*) and C = 0.25 (% *w/v*). Additional experimental runs (*n* = 3) using such parameters were performed for model validation. The resulting nanosuspensions had a particle size of 134.0 ± 0.6 nm (**Fig. ****S1****a**), PDI of 0.179 ± 0.021, physical stability of 72 ± 0 h, and EE of FIN of 90.67 ± 0.08%, which were all in line with the predictions (particle size of 133.83 nm, PDI of 0.161, physical stability of 72 h, and EE of FIN of 91.07%). The DLs of FIN (8.00 ± 0.25%) and CUR (4.43 ± 0.15%) were well aligned with the theoretical values, i.e., 8% for FIN and 4% for CUR. The zeta potential of the optimized nanosuspension was − 0.24 ± 0.24 mV, suggesting its near-neutral state. As shown in **Fig. ****S1****b**, FIN nanoparticles displayed roughly spherical morphology with similar size to that of DLS. As a result, the optimized FIN nanosuspension was used for the subsequent development of nano-embedded microparticle dry powders. It is worth noting that, in this project, the incorporation of CUR as a co-stabilizer within the nanoparticle formulation was crucial in augmenting the stability of the FIN nanosuspension. In fact, unlike FIN, which is widely recognized as a drug, CUR, despite showing promising properties against strokes and other diseases, is generally not classified as a drug by regulatory authorities. Consequently, we have chosen to focus on FIN and employed CUR as an excipient.

**Table 2:** Coded regression equations of response models for optimization of FIN nanosuspension with ANOVA and multiple reliability test (*R*^2^) results

Response	Regression equation	F value	*p* value	*R* ^2^	Adjusted *R*^2^
Particle Size (nm) (Y_1_)	= 147.68 + 17.48 A + 6.28B + 3.26 C + 8.4AC + 7.78BC	128.04	< 0.0001	0.970	0.962
PDI (Y_2_)	= 0.2244–0.0377 A-0.0252B + 0.0321 C + 0.0222AB-0.0218AC	17.28	< 0.0001	0.812	0.765
Physical Stability (hours) (Y_3_)	= 18.00–10.17 A + 17.50B-1.67 C-10.33AB + 6.50AC-1.83BC + 6.00ABC	95.23	< 0.0001	0.974	0.964
EE of FIN (%) (Y_4_)	= 83.22 + 6.10 A + 1.75B-9.36 C-0.6796AB + 3.29AC + 1.13BC	500.23	< 0.0001	0.994	0.992

*Note* Factors BC and C in the equation for Y_3_ were retained to maintain the hierarchy despite being statistically non-significant

### Optimization of FIN Dry Powder formulations

A 2-level, 3-factor full factorial DoE was used to study the influence and interactions of factors [A: mannitol-nanoparticle ratio (w/w); B: atomization flow rate (L/h); and C: feed rate (mL/min)] on the selected properties of the resulting spray-dried nano-embedded powders [Z_1_: redispersibility (RdI); Z_2_: PDI of the reconstituted nanosuspension; and Z_3_: volumetric size of the spray-dried powder] (Table S3). Mannitol was employed as a carrier in spray drying studies due to its promising safety for pulmonary delivery [58, 59]. To preserve the merits of nanoparticles for intranasal delivery, the spray-dried powder should be redispersible to its nanosuspension counterpart, ideally with the same particle size distribution as that freshly prepared from FNP, once in contact with the nasal fluid. Hence, the RdI was set in the range of 0.8–1.2, and the PDI of reconstituted nanosuspension was set as < 0.3. In addition, the particle size of dry powder is a pivotal factor in controlling their nasal deposition profile. Particles less than 5 μm tend to enter the respiratory tract, while particles larger than 20 μm remain in the anterior part of the nose [60, 61]. Only powder with a particle size around 10 μm can effectively deposit in the olfactory region [62, 63]. The target median particle size of the spray-dry powder was therefore set as 10 μm. The levels of each factor were determined based on previous studies [34, 41] and our preliminary data.

The physicochemical properties and volumetric particle size distribution of different FIN dry powder formulations obtained from the DoE were presented in Table 3 and Table S6, respectively. Their RdI varied from 1.02 ± 0.01 to 1.82 ± 0.08, and the PDI of the reconstituted nanosuspensions were close to 0.3. Among different formulations, PF1, PF6, PF7, and PF9 satisfied the pre-set RdI and PDI requirements. The volumetric particle sizes of these dry powder formulations ranged from 4.58 ± 0.57 μm to 10.84 ± 0.47 μm with span *<* 2 (except for PF3). The volumetric median particle size of these formulations, except for PF3 and PF4, was close to the pre-set 10 μm. As shown in Table S6, the EEs of FIN and CUR for all dry powder formulations were all > 90%. The high EE exhibited by the FIN and CUR suggests that they can be effectively encapsulated within nanoparticles without compromising their integrity, even when subjected to elevated temperatures during the spray drying process. The coded regression equations for each response model of FIN dry powder formulations are listed in Table S7. The regression equations for redispersibility and particle size had *p* values < 0.0001, demonstrating a significant relationship between factors and their interactions with the responses. These models are considered reliable as their *R*² values were > 0.90 and agreed with the adjusted R². However, the *p-*value and *R*² values for the PDI regression are 0.715 and 0.200, respectively, indicating that there was no significant factor-response correlation. Hence, the PDI was not included in the numerical optimization process.

**Table 3:** Physicochemical properties of FIN dry powders prepared based on the 3-factor 2-level factorial design (*n* = 3)

Powder Formulation (PF)	Factors with levels			Responses and results		
	A	B	C	RdI(Z_1_)	PDI (Z_2_)	Particle Size D_50_ (µm) (Z_3_)
PF1	10	601	1.5	1.05 ± 0.03	0.299 ± 0.044	8.69 ± 0.21
PF2	10	601	4.5	1.02 ± 0.01	0.347 ± 0.092	9.91 ± 0.28
PF3	10	357	1.5	1.60 ± 0.08	0.338 ± 0.061	5.16 ± 0.62
PF4	10	357	4.5	1.82 ± 0.08	0.310 ± 0.020	4.58 ± 0.57
PF5	4	357	1.5	1.14 ± 0.02	0.303 ± 0.038	8.95 ± 1.14
PF6	4	357	4.5	1.08 ± 0.05	0.293 ± 0.038	10.84 ± 0.47
PF7	4	601	1.5	1.04 ± 0.06	0.285 ± 0.030	8.34 ± 0.28
PF8	4	601	4.5	1.30 ± 0.04	0.305 ± 0.029	7.44 ± 0.49
PF9	7	473	3	1.13 ± 0.03	0.298 ± 0.020	8.38 ± 0.53

A: Mannitol: nano (w/w); B: Atomization gas flow rate (L/h); C: Feed rate (mL/min)

**Fig. 4:**
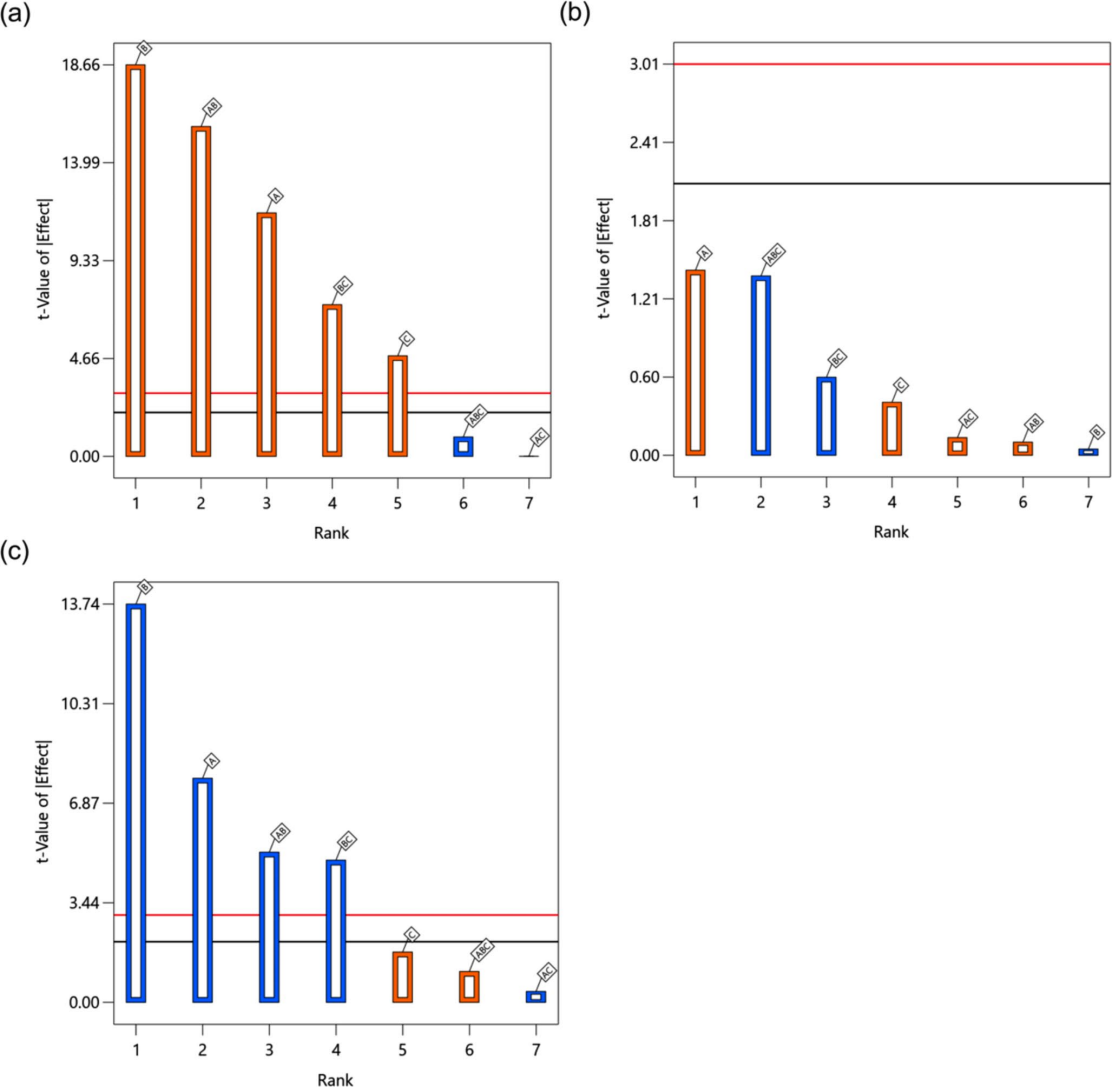
Pareto charts of standardized effects of factors and their interactions on (**a**) redispersibility of the FIN dry powder, (**b**) PDI of the reconstituted nanosuspension and (**c**) volumetric particle size of the FIN dry powder. Factors and interactions that cross the horizontal black line show statistically significant effects on the responses. Blue bar stands for negative effects on the responses, while orange bar stands for positive effects on the responses

The Pareto charts of standardized effects of factors and their interactions on the responses are shown in Fig. 4. All factors and their interactions, except for AC and ABC, showed a positive influence on the RdI of the dry powder. When factor A (mannitol-nanoparticle ratio) increased, the total solute concentration and drying time of nanosuspension droplets would also increase, leading to adverse outcomes on the nanoparticle stability and RdI. The most significant factor affecting RdI was factor B (atomization flow rate) in this study. This was not surprising and could be ascribed to greater shear stresses exerted on the nanoparticle surface during feed atomization. Moreover, a rise in factor C (feed rate) would generate larger droplets, resulting in a longer drying time and stronger thermal stress on the nanoparticles, hence the corresponding increase in RdI. The interaction terms AB and BC also shared the same trend and effect on RdI with their individual factors. Interestingly, all factors and their interactions did not have a significant effect on the PDI of the reconstituted nanosuspension. This implied that these factors had a minimal impact on the uniformity of nanoparticles embedded in the resulting spray-dried powders. Regarding the particle size of the powder, both factors A and B and their interactions AB and BC displayed a negative effect. An increase in factor B would yield smaller droplets and, thus, smaller particles. However, the trend for factor A deviated from literature reports, where particle size increases as solute concentration in feed solution increases [64]. We speculate that the denser dry powders (PF3 and PF4) produced by high solute concentrations with low atomization flow rates were fractured during the dispersion from the nasal device, resulting in a smaller volumetric particle size.

**Fig. 5:**
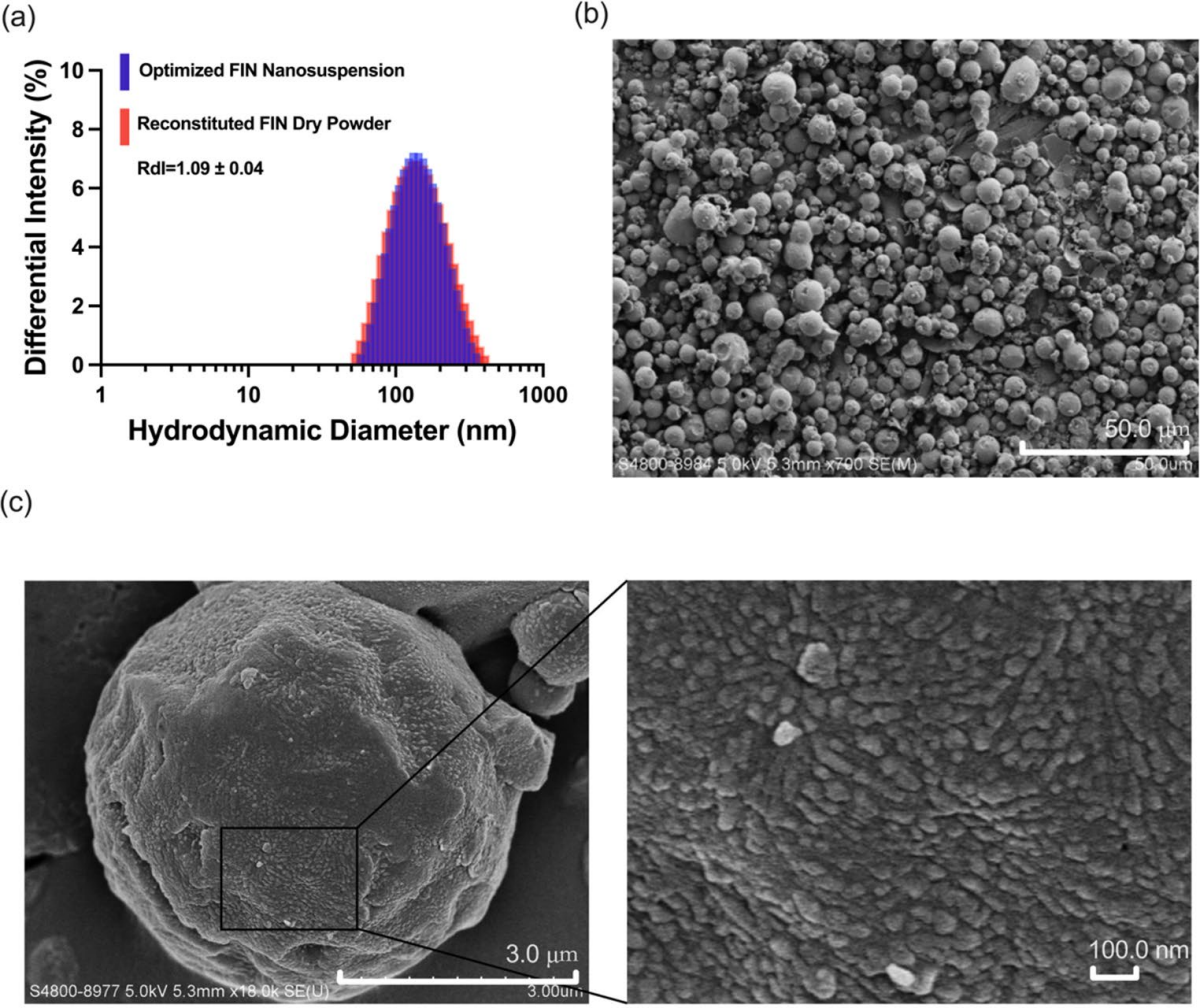
(**a**) Differential intensity with size distribution of the optimized FIN nanosuspension and the reconstituted FIN dry powder for nanosuspension. Scanning electron microscopy (SEM) images of optimized FIN nano-embedded dry powder at (**b**) × 700 magnification (scale bar = 50.0 μm) and (**c**) ×18.0 k magnification (scale bar = 3.0 μm). The embedded nanoparticles were shown in the black frame

The regression models for redispersibility and particle size were simplified by removing non-significant terms (Table 4), and the desirability function was applied to optimize the FIN nano-embedded powder formulation. A moderate level of drug loading can reduce medication dosage. Since an increase in factor A (mannitol-nanoparticle ratio) is correlated with increasing RdI, and it is preferable to minimize the excipients used, the level of A is set as the lowest in the design space. The highest desirability value was achieved when A = 4:1 (*w/w*), B = 407 (L/h), and C = 4.2 (mL/min), and the corresponding predicted RdI and volumetric median particle size of the dry powder were 1.1 and 10 μm, respectively. Model validation was performed for fabricating the FIN dry powder (*n* = 3) using the optimized parameters. The obtained dry powder had a RdI of 1.09 ± 0.04 and a median particle size of 11.31 ± 1.7 μm, congruent with the predicted properties. The PDI (0.272 ± 0.019) of the nanosuspension reconstituted from the dry powders also fulfilled the target requirement. As shown in Fig. 5a, the optimized dry powder could be reconstituted back to nanosuspension without significant changes in particle size distribution compared to the freshly prepared nanosuspension. The volumetric size of the dry powder was 11.31 ± 1.7 μm, which was expected to be effectively deposited in the olfactory region of the nasal cavity. The drug contents of FIN (1.55 ± 0.09%) and CUR (0.68 ± 0.11%) of the dry powder were consistent with the theoretical values (i.e., 1.6% for FIN and 0.8% for CUR).

**Table 4:** Coded regression equations of response models for optimization of FIN dry powders with ANOVA and multiple reliability test (*R*^2^) results

Response	Regression equation	F value	*p* value	*R* ^2^	Adjusted *R*^2^
Redispersibility (Z_1_)	= 1.26 + 0.1150 A + 0.1850B + 0.0475 C + 0.1558AB + 0.0717BC	170.76	< 0.0001	0.977	0.971
Volumetric Particle Size of dry powder (µm) (Z_3_)	= 7.99–0.9046 A-1.61B + 0.2029 C-0.6063AB-0.5738BC	62.77	< 0.0001	0.940	0.925

*Note* Factor C in the equation for Z_3_ was retained to maintain the hierarchy despite being statistically non-significant

### Characterization of the FIN Nano-embedded dry powder formulation

Presented in Fig. 5b and c are the SEM images of the optimized FIN nano-embedded dry powder. The particles exhibited a spherical morphology with embedded nanoparticles (as indicated by the black frame), contributing to their good flowability for ease of powder loading in nasal spraying devices. Considering that a higher aspect ratio of particles could augment powder deposition in the alveolar region, the spherical morphology minimizes the fraction of particles inadvertently deposited in deep lung areas during intranasal application [65]. As seen in the DSC and PXRD profiles (**Figs. S2a and S2b**), all other raw materials, except for PVP, their physical mixture counterpart (which has the same components and composition as the FIN dry powder), and the optimized FIN nano-embedded dry powder were crystalline in nature. The DSC profiles of FIN and CUR revealed an endothermic peak at approximately 127 °C and 183 °C, respectively, consistent with literature data [62, 65]. The DSC traces of the optimized FIN nano-embedded dry powder displayed endothermic peaks at 151∼ 163 °C, corresponding to the melting of the composite particles, similar to their physical mixture counterpart. The DSC and PXRD profiles following 2 months of storage at temperatures of 4, 25, and 40 °C demonstrated consistent traces of optimized FIN nano-embedded dry powder without significant alterations. This observation suggests the stability of optimized FIN nano-embedded dry powder in its crystalline state over the specified storage period. The TGA analysis revealed that approximately 3.01% of residual moisture was present in the optimized dry powders, with a temperature-dependent reduction to 0.91–1.40% after 2 months of storage (**Fig. ****S2****c**). This was not surprising as unbound water in the powder evaporated with a moisture balance. The optimized FIN nano-embedded dry powder exhibited satisfactory stability, with no significant phase transformation or reduction in drug assays detected under various storage conditions. The FTIR spectra of different samples are presented in **Fig. ****S2****d**. A very broad peak can be observed from around 3,600 cm^− 1^ to 3,100 cm^− 1^ in the optimized FIN nano-embedded dry powder. This is likely due to the formation of intermolecular hydrogen bonding between CUR and PVP [66], which improved the stability of the nanoparticles.

Regarding the in vitro drug release, while the optimized FIN nano-embedded dry powder showed rapid and effective redispersion (RdI < 1.2) in simulated nasal fluid, neither FIN nor CUR were detectable by HPLC throughout the 3-hour experiment. The FIN or CUR in the physical mixture in the simulated nasal fluid also could not be detected. It is believed that no drug release occurred in the physical mixture due to its limited solubility, but for the nanosuspension reconstituted from the optimized FIN nano-embedded dry powder, most of the FIN or CUR were well encapsulated and protected within the nanoparticles. The purpose of the physical mixture is to serve as a control and to underscore one of the advantages of nanoparticles, which is their ability to modify physicochemical properties of raw drug, particularly hydrophobic drugs [66]. As shown in **Fig. S3**, the release profile of FIN and CUR from the optimized FIN nano-embedded dry powder outperformed that of the physical mixture in an 80:20 (v/v) solution of simulated nasal fluid and ethanol. This finding supports the notion that nanoparticles can alter the physicochemical properties of hydrophobic compounds (FIN and CUR), thereby expanding their therapeutic potential. The effective encapsulation of FIN or CUR within the nanoparticles also renders the optimized dry powder less susceptible to nasal mucociliary clearance (typically occurring every 10–20 min) [67], making it suitable for nose-to-brain drug delivery.

The nasal deposition studies of the optimized FIN nano-embedded dry powder were conducted using two different nasal anatomical models at varying flow rates of 0, 7.5, and 15 L/min. The flow rates were selected based on the typical breathing patterns pertinent to clinical applications: a flow rate of 15 L/min mimics normal steady breathing [68], 7.5 L/min corresponds to slow inhalation [40], and 0 L/min represents an absence of breathing or a breath-holding scenario [14, 69]. All nasal models were connected to an NGI to simultaneously study powder deposition in the nose and the respiratory tract.

**Fig. 6:**
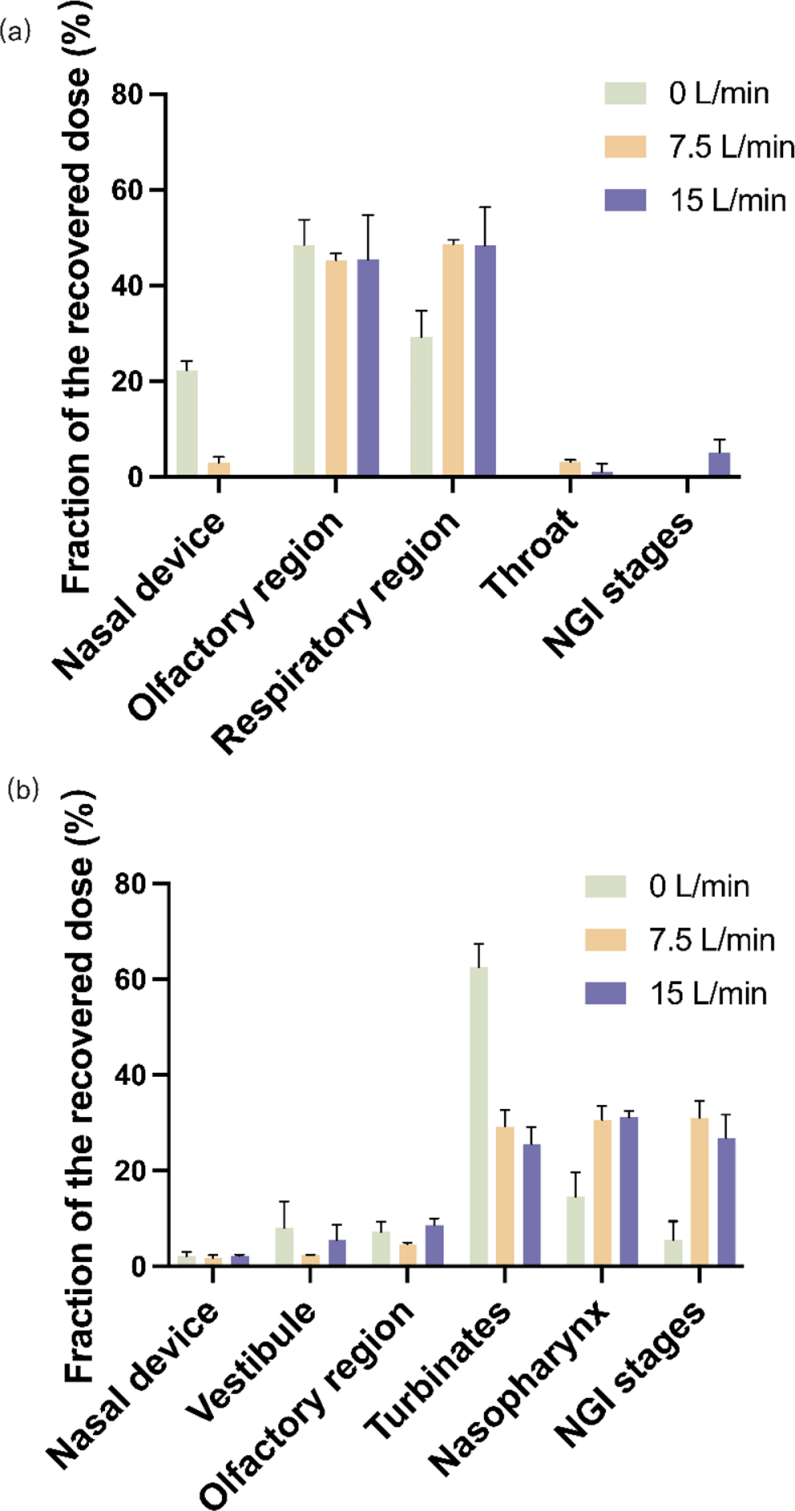
Fraction of recovered dose on nasal model regions, nasal device and NGI stages of optimized FIN nano-embedded dry powder at inspiratory flow rates of 0, 7.5 and 15 L/min using (**a**) the 3D-printed nasal cast and (**b**) the AINI model

The powders demonstrated excellent dispersibility upon actuation of the nasal device, with the nasal device fraction consistently below 5% (Fig. 6), except for the 3D-printed nasal cast at 0 L/min flow rate. These findings suggest that the nasal device is generally effective for administering powder formulations to the nasal cavity. The high deposition in the nasal device (~ 20%) observed for the 3D-printed nasal cast at 0 L/min flow rate may be attributed to the powder backflow from the respiratory region of the cast to the exterior of the nasal device due to insufficient energy from the flow.

The olfactory pathway is an important route for nose-to-brain delivery [70, 71]. In the 3D-printed nasal cast (Fig. 6a), the deposition fractions of optimized FIN nano-embedded dry powders in the olfactory region were 45.4% for 15 L/min, 45.2% for 7.5 L/min, and 48.5% for 0 L/min. Hence, as high as 45% of the optimized FIN nano-embedded dry powder can deposit in the olfactory region. In the AINI model (Fig. 6b), the deposition fractions of optimized FIN nano-embedded dry powder in the olfactory region were 8.6% for 15 L/min, 4.7% for 7.5 L/min, and 7.3% for 0 L/min. Aside from the olfactory region being the primary focus for nose-to-brain delivery, the highly vascularized respiratory region also warrants attention, as drug can be absorbed in this area for systemic delivery. In addition, parts of the turbinate region innervated by the trigeminal nerve can transport the drug from the nose to the brain via the trigeminal pathway [5, 69]. In the 3D-printed nasal cast, the deposition fractions of optimized FIN nano-embedded dry powder in the respiratory region were 48.3% for 15 L/min, 48.5% for 7.5 L/min, and 29.2% for 0 L/min. In the AINI model, the deposition fractions of optimized FIN nano-embedded dry powder in the respiratory region (sum of nasopharynx, turbinates, and vestibule) were 62.3% for 15 L/min, 62.3% for 7.5 L/min, and 85.0% for 0 L/min. In detail, the deposition fractions of optimized FIN nano-embedded dry powder in the turbinate region were 25.6% for 15 L/min, 29.2% for 7.5 L/min, and 62.5% for 0 L/min.

The discrepancy in regional deposition profiles between the 3D-printed nasal cast and the AINI was not surprising and can be ascribed to the differences in the design and construction materials of these models. Since the olfactory region of the 3D-printed nasal cast has a larger surface area, the deposition fraction was higher in this model. Apart from the design, the 3D-printed nasal cast was made of polylactic acid, while the AINI model was fabricated from stainless steel. The adhesion capabilities, texture, and potential impact of surface charges on dry powders may exhibit variations. In the present study, the optimized FIN nano-embedded dry powders displayed a greater propensity to adhere to plastic surfaces. Powders in the olfactory region of the AINI model may have unintentionally fallen into the respiratory region (especially during the disassembly of the AINI), resulting in a lower deposition fraction. The in vitro-in vivo correlation of drug deposition using these nasal models should be further investigated.

It is worth noting that the inspiratory flow rate may vary among patients of different ages and disease states. Therefore, a holistic evaluation of how the inspiratory flow rate may influence the nasal deposition is desired. Perkušić et al. found that an increased inspiratory flow rate from 0 L/min to 60 L/min was associated with reduced drug deposition in the olfactory region [14], while Rigaut et al. found that flow rate (0, 15, and 60 L/min) was not a significant influencing factor for olfactory deposition [68]. In this study, the deposition fraction of the olfactory region was independent of the flow rate for the optimized FIN powder formulation under different flow rates in two different nasal models (Fig. S4). The reason for this observation could be due to the restricted range of inspiratory flow rate (0–15 L/min) studied. Besides, the manufacturer of the Unidose Nasal Spray Device (Aptar Pharma) has suggested that simultaneous actuation of the nasal device and inspiration is not required because the nasal device can generate sufficient air pressure for powder dispersion [68]. Hence, the optimized powder formulation is suitable for patients with different inspiratory flow rates.

### In Vitro cytotoxicity studies of the optimized FIN Nano-embedded dry powder formulation

**Fig. 7:**
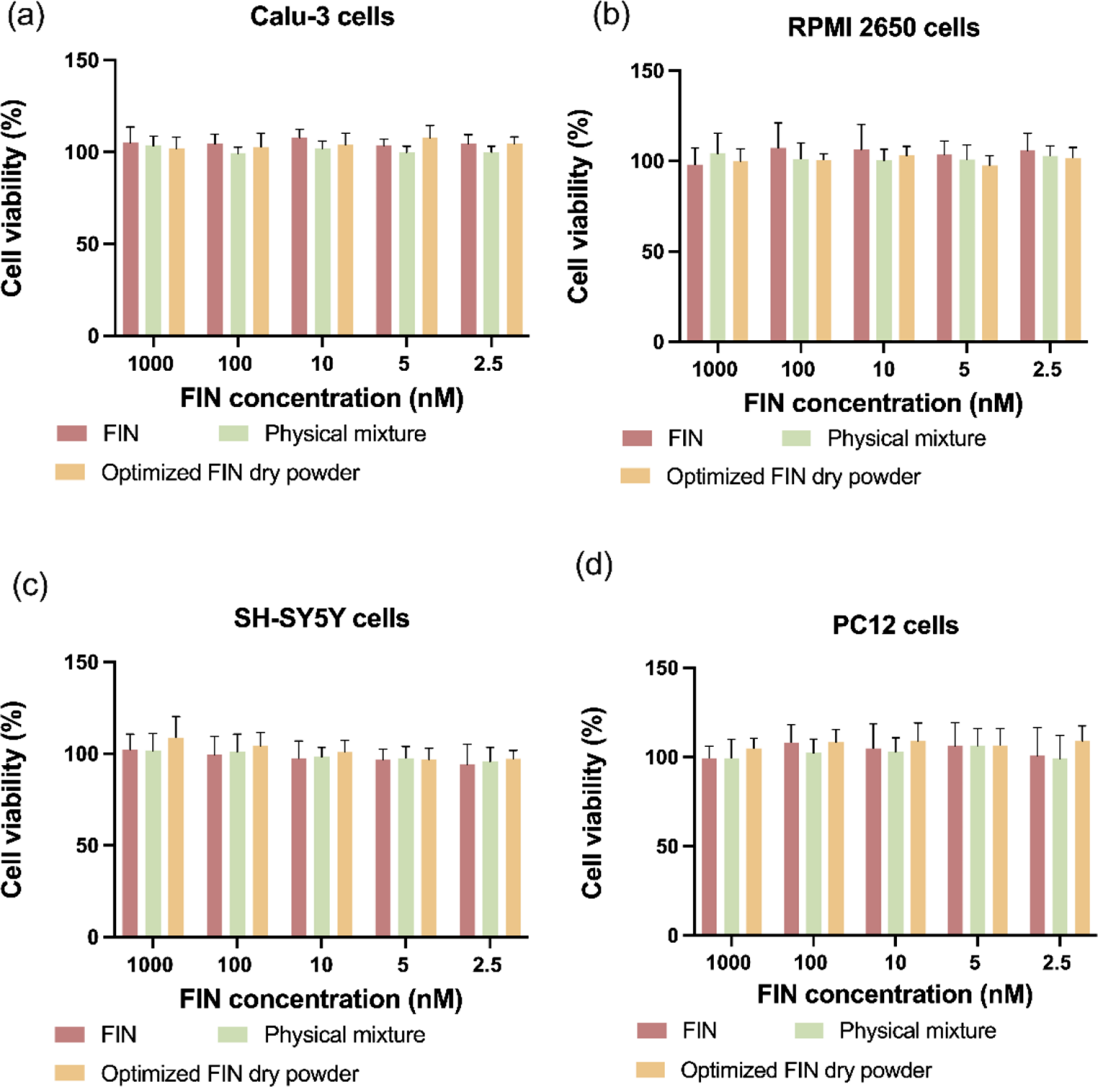
The cytotoxicity of raw FIN, the physical mixture of the formulation components, and reconstituted FIN dry powder for nanosuspension in (**a**) Calu-3 cells, (**b**) RPMI 2650 cells, (**c**) SH-SY5Y cells, and (**d**) PC 12 cells at the FIN concentration ranged 2.5–1,000 nM

The cytotoxicity of FIN nanosuspension reconstituted from the optimized dry powder was evaluated in various cell lines. SH-SY5Y and PC 12 cells were used as neuronal cell models, while RPMI 2650 and Calu-3 were used as nasal epithelial cell models [72]. In addition, the cytotoxicity of raw FIN and a physical mixture of the optimized powder formulation composition were also investigated for comparison purposes. The concentration range studied (2.5–1,000 nM) was chosen based on literature reports and clinical pharmacokinetics of FIN [73, 74]. As presented in Fig. 7, the cell viability for all groups was around 100%, with no significant difference observed. This result indicates that the formulation has an acceptable in vitro safety profile.

**Fig. 8:**
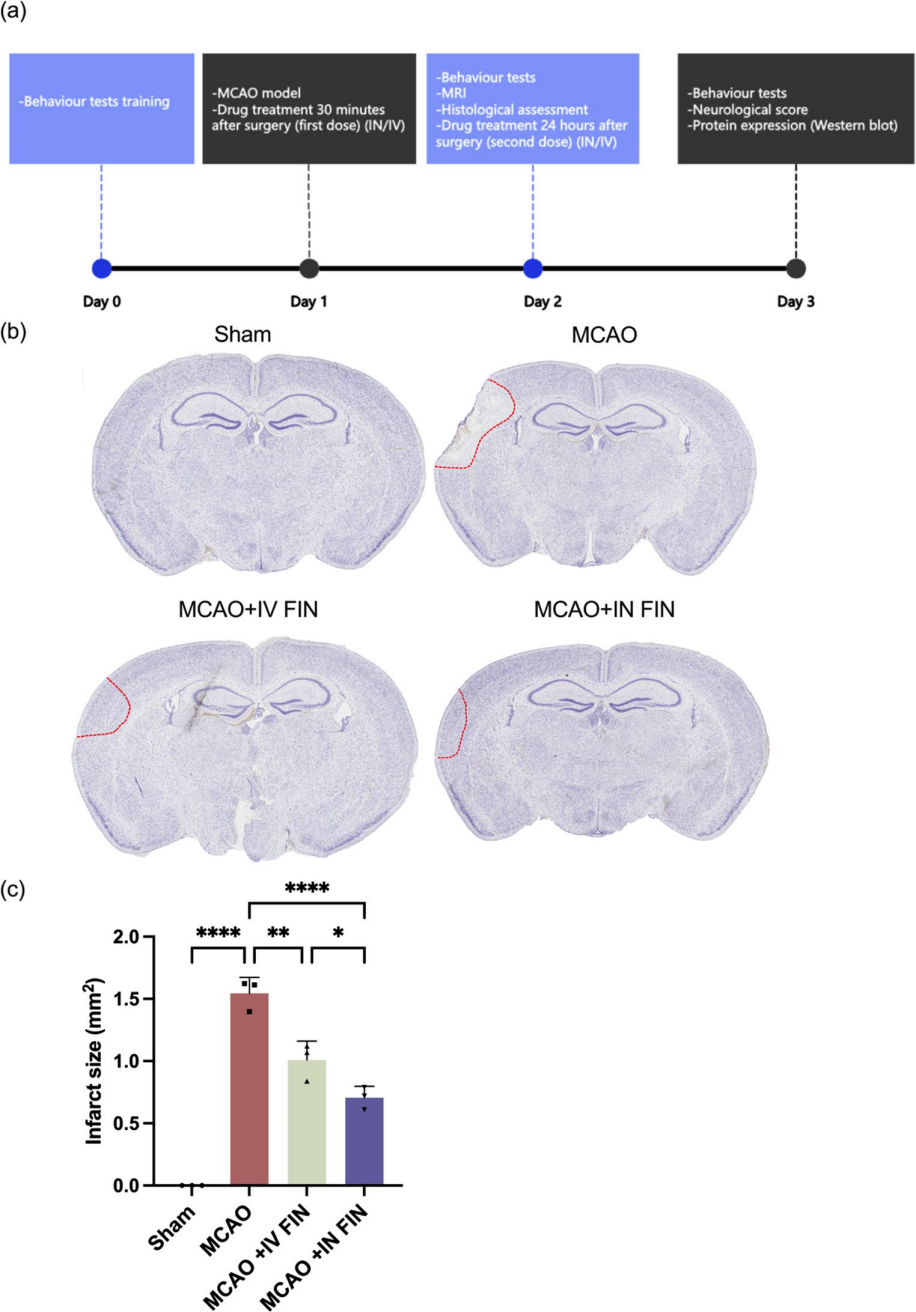
(**a**) Experimental scheme of the in vivo animal experiments. (**b**) Representative images of the brain slices stained by Nissl staining solution on day 2. The infarct regions were marked by red line. (**c**) The infarct volumes of brain in each group (*n* = 3). Data are presented as means ± SD, * *p* < 0.05, ** *p* < 0.01, **** *p* < 0.0001

### In vivo neuroprotective effects of the optimized FIN Nano-embedded dry powder formulation

**Fig. 9:**
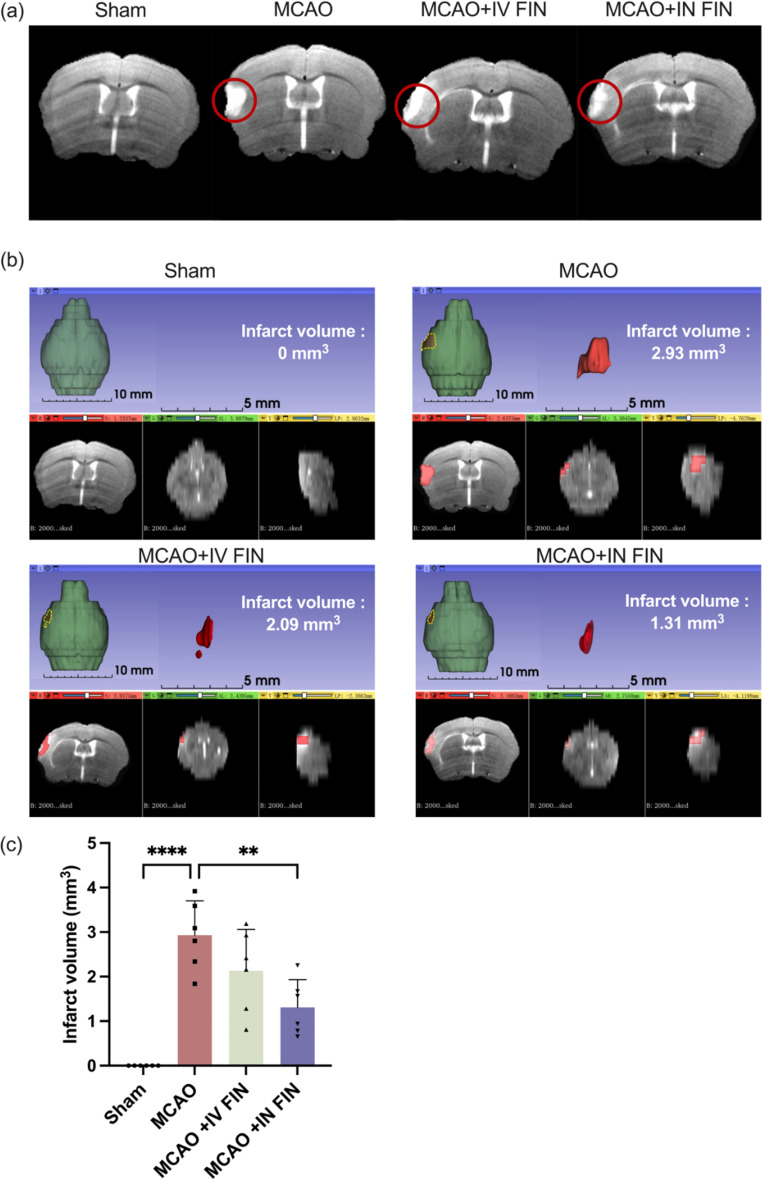
(**a**) Representative images of brain MRI in each group. The infarct region is circled by red line. (**b**) Representative images of the infarct core (upper left) and three-dimensional images of brain infarction (upper right). The coronal section, horizontal section, and sagittal section are shown from bottom left to right in MCAO mice treated with 0.9% saline intranasally, reconstituted FIN dry powder for nanosuspension intravenously, and reconstituted FIN dry powders for nanosuspension intranasally on day 2. (**c**) The infarct volumes of brain in each group (*n* = 6). Data are presented as means ± SD, ** *p* < 0.01, **** *p* < 0.0001

A schematic diagram of the study design of in vivo neuroprotective effects is depicted in Fig. 8a. FIN nanosuspension was reconstituted from the optimized dry powder and separately delivered to the mice with MCAO surgery via intravenous (IV) and intranasal (IN) administration. To assess the neuroprotective effect with greater clinical relevance, the tested drugs were administrated 30 min after stroke surgery. As shown in Fig. 8b, the brain slices were stained with Nissl solutions on day 2 and the infarct regions were marked by the red line. The infarct size was significantly reduced to 1.01 ± 0.12 mm^2^ in the IV-treated group compared with the untreated MCAO group (1.54 ± 0.10 mm^3^). Remarkably, the infarct size was further reduced to 0.71 ± 0.07 mm^2^ in the IN-treated group (Fig. 8c**)**. To precisely measure the cerebral infarct size, we also employed the 9.4T animal magnetic resonance imaging system (MRI) 24 h post-treatment. Figure 9a shows the mouse brain MRI images with clear cerebral infarction after MCAO surgery in the left hemispheres, with an average infarct volume of 2.93 ± 0.71 mm^3^. The IV-treated mice had a lower infarct volume of 2.13 ± 0.85 mm^3^, while the IN-treated group gave a significant reduction to 1.31 ± 0.57 mm^3^ (Fig. 9b and c).

**Fig. 10:**
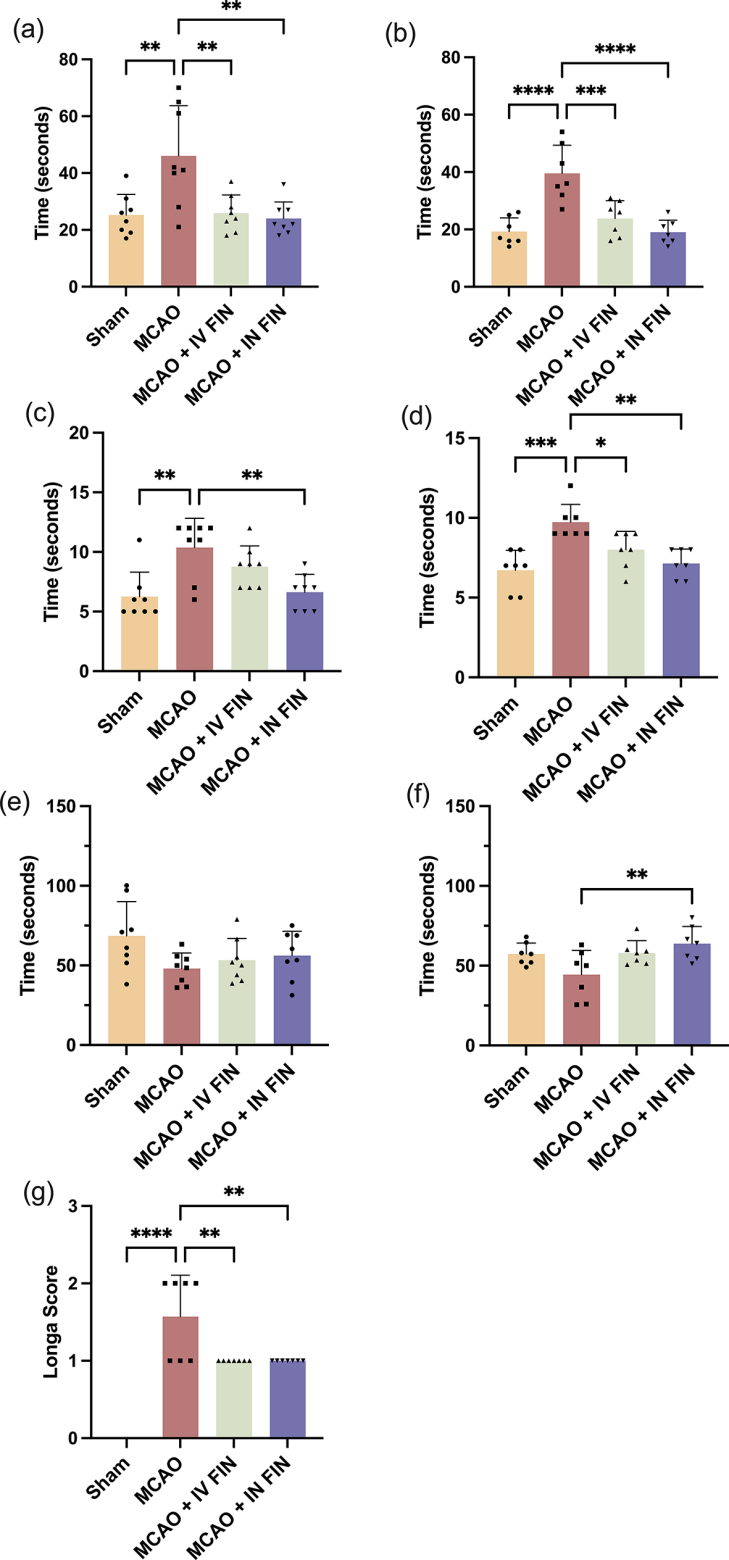
The neurological functional assessments of mice. Adhesive removal tests on day 2 (**a**) and day 3 (**b**), Cross beam tests on day 2 (**c**) and day 3 (**d**), Rotarod tests on day 2 (**e**) and day 3 (**f**), Neurological deficit evaluation by Longa scores on day 3 (**g**). Data are presented as means ± SD, *n* = 8 for day 2 and *n* = 7 for day 3. * *p* < 0.05, ** *p* < 0.01, *** *p* < 0.001 and **** *p* < 0.0001

The neuroprotective effects were also evaluated using the adhesive removal test, balance beam test, rotarod test, and the Longa score. As expected, the time required for the mice to remove the adhesive tapes from their bilateral paws after MCAO surgery was significantly increased due to the neurological deficit after acute ischemia imposed by the MCAO surgery (Fig. 10a, b). However, the time required for MCAO mice with IV and IN treatment to remove the adhesive tapes decreased significantly compared to untreated MCAO mice on day 2 and day 3 (Fig. 10a and b) after stroke surgery. Notably, the effect on the IN-treated group on day 3 was more significant than that of the IV-treated group, as evidenced by the smaller *p*-value. Likewise, MCAO mice treated with reconstituted FIN nanosuspension intranasally required less average time to cross the beam compared to untreated MCAO mice and IV-treated MCAO mice (Fig. 10c and d**)**. For the rotarod test, however, the time spent on the rod for treated groups was not statistically significant from that in the untreated group on day 2 (Fig. 10e), possibly due to the sensitivity of the rotarod test for the stroke model [75]. Nevertheless, a statistically significant difference (*p* < 0.01) was observed between the MCAO + IN FIN and MCAO groups on day 3 (Fig. 10f). The Longa scores of mice in both treatment groups significantly decreased compared to the untreated MCAO group (Fig. 10g). Taken together, these results suggest that the IN administration outperformed IV administration in terms of alleviating neurological deficits after a stroke.

**Fig. 11:**
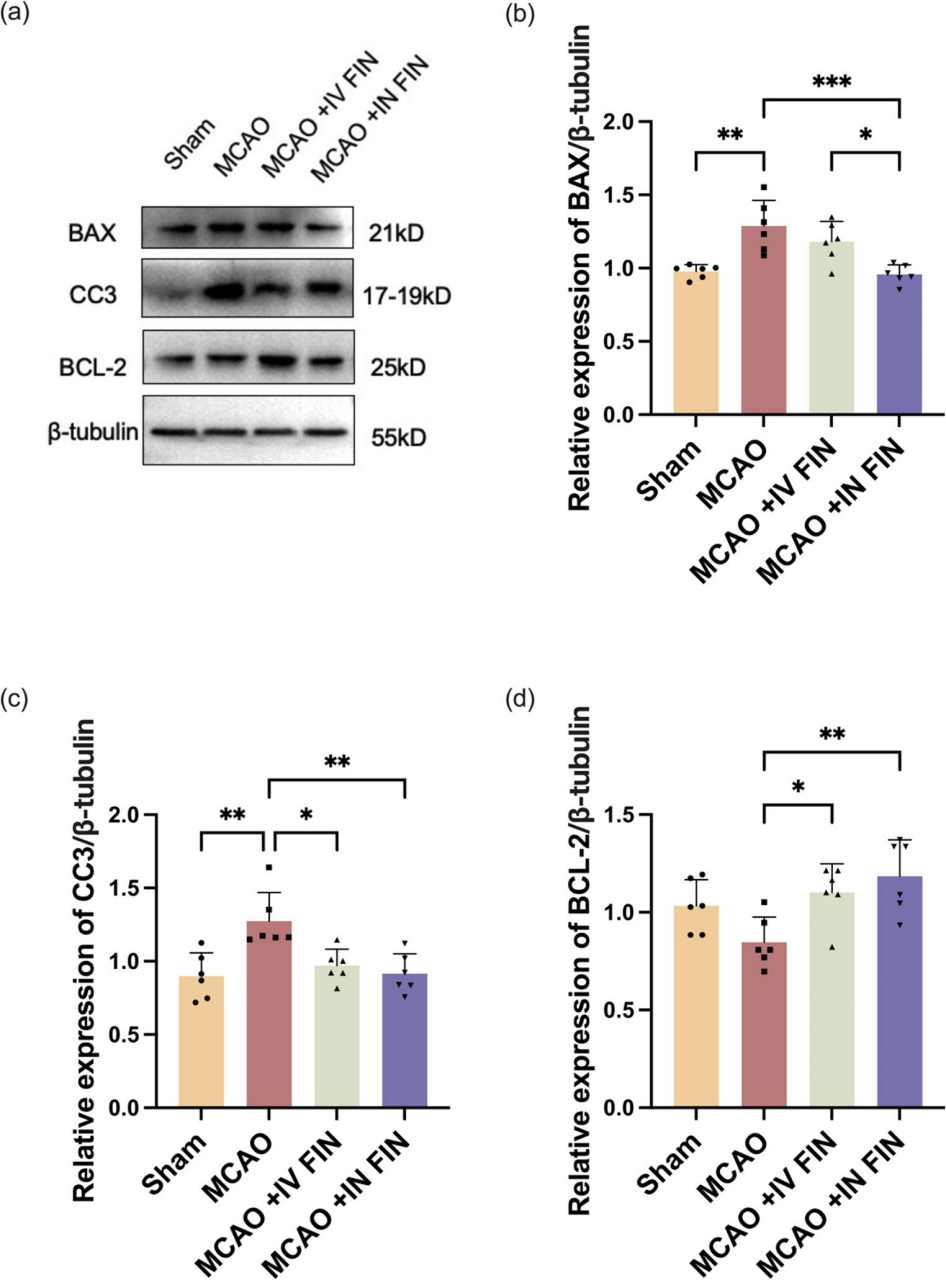
(**a**) Representative western blot images showing the protein expression of BAX, CC3 and BCL-2 in each group after treatment on day 3. Quantification of the Western blot results showing the relative expression of (**b**) BAX (**c**) CC3 and (**d**) BCL-2 to β-tubulin in each group. Data are presented as means ± SD, *n* = 6, * *p* < 0.05, ** *p* < 0.01, *** *p* < 0.001

Furthermore, a western blot analysis was performed on post-stroke mouse brains to understand the neuroprotective mechanism. Expression of pro-apoptotic proteins BAX and CC3, and the anti-apoptotic protein BCL-2 in the peri-infarct tissue of our MCAO stroke model were determined [51]. This was due to reports that FIN could increase the expression of BCL-2 and decrease the expressions of CC3 and BAX [76, 77]. As shown in Fig. 11, administration of the reconstituted FIN dry powder for nanosuspension by IV and IN routes showed significant neuroprotective effects by reducing the expression of apoptosis proteins CC3 and BAX and increasing the expression of anti-apoptotic protein BCL-2. Of note, expression of BAX in the peri-infarct tissue was statistically significantly lower in IN-treated mice compared to that in IV-treated mice. BCL-2 expression was also numerically higher in IN-treated mice relative to IV-treated mice despite the lack of statistically significant differences. Nevertheless, these results highlight that IN delivery of FIN nanoparticles was more effective in reducing infarct size in the peri-infarct tissue than IV delivery.

It is noteworthy that the neuroprotective effects could be achieved after single-dose IN administration. The non-invasive and convenient IN administration method also presents a unique treatment modality where FIN nanoparticles (in form of dry powder or reconstituted nanosuspension) can be timely administered by ambulance staff or caregivers to patients experiencing acute ischemic stroke symptoms before hospital admission. This strategy can attenuate neuronal injury in the hyperacute phase of ischemic stroke between stroke onset and in-hospital treatment procedures and promote post-stroke functional recovery. Whilst cardiovascular adverse effects such as bradycardia and atrioventricular blockade have been observed in patients on chronic oral FIN treatment for multiple sclerosis [78], we do not anticipate significant safety issues arising from single-dose IN nanoparticle administration due to its excellent cytocompatibility after single-dose treatment.

A constraint of the current study is that mice were administered IN with reconstituted FIN nanosuspensions instead of the optimized FIN nano-embedded dry powder as precise powder administration to the small nares of rodents is challenging. Nonetheless, this study provides proof-of-concept evidence of the neuroprotective effects by FIN nanoparticles. It is anticipated that the administration of dry powders would offer prolonged nasal retention and thus further enhance nose-to-brain FIN transport and neuroprotective efficacy. On-going studies are attempting to devise an appropriate technique for administering powder into the nares of rodents in rodent MCAO models.

The nasal anatomy exhibits notable differences between humans and rodents, exemplified by the contrasting ratio of surface area to luminal volume in their respective nasal cavities [79]. Specifically, while the ratio in rats stands at 3,350 mm^2^/cm^3^, it is markedly lower in humans at 820 mm^2^/cm^3^ [80]. This discrepancy in the ratio of olfactory mucosa to overall nasal volume in rodents may result in an overestimation of drug efficacy, potentially causing translational failures in clinical trials. Mitigating this issue requires meticulous selection of an appropriate animal model (e.g., non-human primates) and precise determination of intranasal dosage during in vivo experiments. Non-human primates, due to their anatomical resemblance and olfactory region occupancy akin to humans, have emerged as a promising model for investigating nose-to-brain drug delivery [81]. Despite their advantageous physiological parallels, constraints such as cost, accessibility, and ethical considerations have prompted the utilization of rodents in this study to establish initial feasibility, while acknowledging the need for subsequent investigations. Adjusting the intranasal dosage for rodents based on the body surface area ratio to humans as defined by Paget and Barnes can aid in addressing this concern [82, 83]. The selection of a dosage regimen mirroring the clinical human dose, specifically 1 mg/kg of FIN, in this study underscores an effort to enhance the translational relevance of preclinical outcomes to clinical effectiveness assessments. Moreover, following a stroke, the basal ganglia, parietal lobe, and frontal lobe are the most affected anatomical regions [84]. While it has been demonstrated that nasal-to-brain delivery can transport drugs to the brain parenchyma, the substantial distance between the olfactory region and these specific areas means that drugs must traverse a considerable distance (approximately in millimeters) for drug delivery to reach the intended target site [85–87]. Careful consideration of this spatial relationship is crucial, as enhancing the specificity of drug delivery to these regions can substantially augment treatment efficacy. Future advancements in our FIN nanoparticle, particularly through tailored modifications to enhance targeting capabilities, hold promise for optimizing treatment outcomes in these contexts.

The dissolution behavior of the optimized FIN nano-embedded dry powder in simulated nasal fluid has been validated in this study. However, it is important to note that although simulated nasal fluid was employed in this investigation to replicate the nasal environment, there may be discrepancies with the actual conditions in the human nasal cavity, particularly concerning aspects such as nasal mucus, enzymatic breakdown, and mucociliary clearance. Nasal mucus comprises a liquid layer known as the “periciliary liquid,” overlaid by a more viscous gel, along with a surfactant layer that helps distribute mucus across the epithelial surface [88]. Enzymes present in the nasal mucus have the potential to degrade drugs [88]. Additionally, the particle transport facilitated by mucociliary clearance could impede the absorption of medications in aqueous formulations, with a duration estimated to be around 12–15 min [89]. These factors may impact the dissolution behavior of drugs in the human nasal cavity and require further investigation. In addition, further exploration of its in vivo drug release from nanoparticle in the brain is warranted. To this end, drug targeting efficiency, direct transport percentage, and pharmacokinetic analysis in in vivo studies can serve as valuable tools for quantitatively predicting the biofate of the intranasally administered optimized FIN nano-embedded dry powder in the brain. Moreover, future investigations should encompass dose-response studies of intranasally administered optimized FIN nano-embedded dry powder, while being mindful of potential overestimation issues. The ultimate goal is to identify an optimal powder dose that can serve as an effective and rapid neuroprotective therapy after an acute ischemic stroke.

## Conclusions

A FIN nano-embedded dry powder formulation for intranasal application was developed using a full factorial design of experiments. The optimized FIN nanosuspension had a particle size of 134.0 ± 0.6 nm, a satisfactory PDI, and acceptable stability. The nanosuspension was then spray-dried into a nano-embedded microparticles dry powder with the aid of mannitol. The optimized dry powder exhibited excellent redispersibility (RdI = 1.09 ± 0.04) and good drug deposition in the olfactory region. The deposition fractions in the olfactory region were found to be independent of the nasal inspiratory flow rate, rendering it suitable for patients with different clinical conditions. It also had acceptable safety profiles in both nasal and brain cell models. Improved behavioral test results, reduced infarct volume, altered expressions of anti-apoptotic and pro-apoptosis proteins were observed following IN administration of the reconstituted FIN nanosuspension in a MCAO mouse model. This study demonstrates the neuroprotective effects of IN and IV FIN nanoparticles, with IN administration showing superiority for ischemic stroke management. Investigations into dose-response effects and pharmacokinetics of the FIN nano-embedded dry powder via IN and IV administrations are ongoing to further optimize its efficacy.

## Electronic supplementary material

Below is the link to the electronic supplementary material.

Supplementary Material 1

Supplementary Material 2

## Data Availability

The datasets generated during and/or analysed during the current study are available from the corresponding author on reasonable request.

## Abbreviations

AINI: Alberta Idealised Nasal Inlet
ATCC: American Type Cultural Collection
BAX: B-cell lymphoma 2 associated X
BCL-2: B-cell lymphoma 2
CC3: cleaved Caspase-3
CLT: cholesterol
CQA: critical quality attributes
CUR: curcumin
DL: drug loading
DLS: dynamic light scattering
DMEM/F12: Dulbecco’s Modified Eagle Medium/Nutrient Mixture F-12
DMEM: Dulbecco’s Modified Eagle’s Medium
DSC: differential scanning calorimetry
DoE: Design of Experiment
EE: encapsulation efficiency
EtOH: ethanol
F-12K: Kaighn’s Modification of Ham’s F-12
FBS: fetal bovine serum
FIN: fingolimod
FTIR: Fourier-transform infrared spectroscopy
IN: intranasal
IV: intravenous
MCAO: middle cerebral artery occlusion
MEM: Minimum Essential Medium
MeOH: methanol
MIVM: multi-inlet vortex mixer
MRI: magnetic resonance imaging
NGI: next generation impactor
PBS: phosphate-buffered saline
PDI: polydispersity index
PVP: Polyvinylpyrrolidone
PXRD: powder x-ray diffractometry
RdI: redispersibility index
Re: Reynold number
S.D.: standard deviation
TEM: transmission electron microscopy
TFA: trifiuoroacetic acid
TGA: thermogravimetric analysis
UPW: ultra-purified water
