# Supplementary material for: Rational development of fingolimod nano-embedded microparticles as nose-to-brain neuroprotective therapy for ischemic stroke
Source: Drug Deliv Transl Res. 2024 Nov 1;15(6):2022–47. doi: 10.1007/s13346-024-01721-8 (PMC12037672; doi:10.1007/s13346-024-01721-8)
Supplement: Supplementary file 2 — Supplementary Material 2 [file 13346_2024_1721_MOESM2_ESM.docx]

**Appendix A. Supplementary Data**

**Rational Development of Fingolimod Nano-embedded Microparticles as Nose-to-Brain Neuroprotective Therapy for Ischemic Stroke**

Xinyue Zhang ^a,b^, Guangpu Su ^c^, Zitong Shao ^a,b^, Ho Wan Chan ^a^, Si Li ^a,b^, Stephanie Chow ^a^, Chi Kwan Tsang ^c^, Shing Fung Chow ^a,b,*^

^a^ Department of Pharmacology and Pharmacy, Li Ka Shing Faculty of Medicine, The University of Hong Kong, Pokfulam, Hong Kong SAR, China

^b^ Advanced Biomedical Instrumentation Centre, Hong Kong Science Park, Shatin, Hong Kong SAR, China

^c^ Clinical Neuroscience Institute and Department of Neurology, The First Affiliated Hospital of Jinan University, Guangzhou, Guangdong Province, China

*^*^* *Corresponding author*

**Table S1**

The gradient mode of FIN and CUR in HPLC.

| **Time (min)** | **Mobile phase A (% v/v)** | **Mobile phase B (% v/v)** |
| --- | --- | --- |
| 0 | 23 | 77 |
| 10 | 15 | 85 |
| 11 | 15 | 85 |
| 12 | 23 | 77 |
| 17 | 23 | 77 |

**Table S2**

A 3-factor, 2-level full factorial design for the preparation of FIN nanosuspension

| **Factors: Formulation Parameters** | | **Levels** | | |
| --- | --- | --- | --- | --- |
|  |  | **-1** | **0** | **+1** |
| A: Initial FIN concentration (mg/ml)  B: CLT: FIN (w/w)  C: Concentration of PVP solution (% w/v) | | 5  0.5  0.25 | 7.5  1  0.625 | 10  1.5  1 |
| **Responses (Y)** | **Goals** | **Requirements** | | |
| Y_1_: Particle Size of nanoparticle (nm)  Y_2_: PDI  Y_3_: Physical Stability (hours)  Y_4_: Encapsulation Efficiency of FIN (%) | In range  Minimize  Maximize  Maximize | 0-200 nm  < 0.3  > 24 hours  > 90 % | | |

**Table S3**

A 3-factor, 2-level full factorial design for the fabrication of FIN dry powder.

| **Factors: Formulation Parameters** | | **Levels** | | |
| --- | --- | --- | --- | --- |
|  |  | **-1** | **0** | **+1** |
| A: Mannitol: nano (w/w)  B: Atomization gas flow rate (L/h)  C: Feed rate (ml/min) | | 4  357  1.5 | 7  473  3 | 10  601  4.5 |
| **Responses (Y)** | **Goals** | **Requirements** | | |
| Z_1_: Redispersibility  Z_2_: PDI  Z_3_: Volumetric particle size of dry powder | In range  Minimize  Target | 0.8 - 1.2  < 0.3  10 µm | | |

**Table S4**

Initial trials on FIN nanosuspension fabrication by FNP (initial FIN concentration = 5 mg/mL; n = 3).

| **Trial** | **Stabilizer** | **Co-stabilizer** | **Particle size (nm)** | **Physical stability** |
| --- | --- | --- | --- | --- |
| 1 | PVP  [0.5% (w/v)] | / | 1290.5±20.7 | 5 minutes |
| 2 | TPGS  [FIN-CUR-TPGS ratio = 1:1:1 (w/w)] | / | NA^*^ | NA^^^ |
| 3 | TPGS  [FIN-CUR-TPGS ratio = 1:1:1 (w/w)] | CLT  [FIN -CLT ratio = 5:1 (w/w)] | NA^*^ | NA^^^ |
| 4 | PVP  [0.5% (w/v)] | CLT  [FIN-CUR-CLT ratio = 2:1:2 (w/w)] | 228.0±10.3 | 1 h |
| 5 | TPGS  [FIN-TPGS ratio = 1:1 (w/w)] | CLT  [FIN -CLT ratio = 5:1 (w/w)] | NA^*^ | NA^^^ |

NA^*^: The particle size of nanoparticle was not detected under the detection by dynamic light scattering.

NA^^^: The physical stability of nanoparticle was not available due to the missing particle size data.

**Table S5**

Coded regression equations for probing the relationship between formulation parameters and CQAs of FIN nanosuspensions with ANOVA and multiple reliability test (*R*^2^) results.

| **Response** | **Regression equation** | **F value** | **p value** | **R^2^** | **Adjusted R^2^** |
| --- | --- | --- | --- | --- | --- |
| Particle Size (nm) (Y_1_) | *=*147.68+17.48A+6.28B+3.26C-1.92AB+8.4AC+7.78BC-1.72 ABC | 148.33 | < 0.0001 | 0.983 | 0.976 |
| PDI (Y_2_) | =0.2244-0.0377A-0.0252 B+0.0321C+0.0222AB-0.0218 AC-0.0127BC | 14.41 | < 0.0001 | 0.849 | 0.790 |
| Physical Stability (hours) (Y_3_) | =18.00-10.17A+17.50B-1.67C-10.33AB+6.50AC-1.83BC+6.00ABC | 95.23 | < 0.0001 | 0.974 | 0.964 |
| EE of FIN (%) (Y_4_) | =83.22+6.10A+1.75B-9.36C-0.6796AB+3.29AC+1.13BC-0.4029ABC | 497.62 | < 0.0001 | 0.995 | 0.993 |

**Table S6**

Volumetric particle size distribution of FIN dry powder formulations and EE of FIN/CUR in FIN dry powder formulations. The powders were dispersed using the nasal powder device at 15 L/min and the particle size distribution was measured by laser diffraction (n=3).

| **Powder**  **Formulation**  **(PF)** | **D_10_ (µm)** | **D_50_ (µm)** | **D_90_ (µm)** | **Span** | **EE of FIN (%)** | **EE of CUR (%)** |
| --- | --- | --- | --- | --- | --- | --- |
| PF1 | 2.27 ± 0.34 | 8.69 ± 0.21 | 17.76 ± 0.12 | 1.78 ± 0.03 | 96.93 ± 0.01 | 99.99 ± 0.00 |
| PF2 | 2.97 ± 0.11 | 9.91 ± 0.28 | 19.53 ± 0.88 | 1.67 ± 0.05 | 99.99 ± 0.00 | 99.99 ± 0.00 |
| PF3 | 1.51 ± 0.18 | 5.16 ± 0.62 | 12.43 ± 2.17 | 2.10 ± 0.14 | 99.99 ± 0.00 | 99.99 ± 0.00 |
| PF4 | 1.49 ± 0.25 | 4.58 ± 0.57 | 10.67 ± 2.15 | 1.99 ± 0.19 | 91.03 ± 0.01 | 99.99 ± 0.00 |
| PF5 | 2.27 ± 0.04 | 8.95 ± 1.14 | 19.02 ± 2.36 | 1.87 ± 0.03 | 97.38 ± 0.00 | 99.99 ± 0.00 |
| PF6 | 3.20 ± 0.11 | 10.84 ± 0.47 | 24.44 ± 3.61 | 1.95 ± 0.25 | 98.34 ± 0.00 | 99.99 ± 0.00 |
| PF7 | 2.72 ± 0.17 | 8.34 ± 0.28 | 17.04 ± 0.23 | 1.72 ± 0.06 | 99.20 ± 0.00 | 99.99 ± 0.00 |
| PF8 | 2.29 ± 0.30 | 7.44 ± 0.49 | 15.47 ± 1.38 | 1.77 ± 0.04 | 99.99 ± 0.00 | 99.99 ± 0.00 |
| PF9 | 2.39 ± 0.07 | 8.38 ± 0.53 | 16.87 ± 0.98 | 1.73 ± 0.03 | 99.99 ± 0.00 | 99.99 ± 0.00 |

**Table S7**

Coded regression equations for probing the relationship between formulation and process parameters and CQAs of FIN powder with ANOVA and multiple reliability test (*R*^2^) results.

| **Response** | **Regression equation** | **F value** | **p value** | **R^2^** | **Adjusted R^2^** |
| --- | --- | --- | --- | --- | --- |
| Redispersibility (Z_1_) | =1.26+0.1150A+0.1850B+0.0475C+0.1558AB+0.0000AC+0.0717BC-0.0092ABC | 115.11 | < 0.0001 | 0.978 | 0.970 |
| PDI (Z_2_) | =0.3100+0.0135A-0.0005B+0.0039C+0.0010AB+0.0013AC-0.0057BC-0.0131ABC | 0.6429 | 0.7154 | 0.200 | -0.111 |
| Volumetric Particle Size of dry powder (µm) (Z_3_) | =7.99-0.9046A-1.61B+0.2029C-0.6063AB-0.0438AC-0.5738BC+0.1246ABC | 43.39 | < 0.0001 | 0.944 | 0.922 |


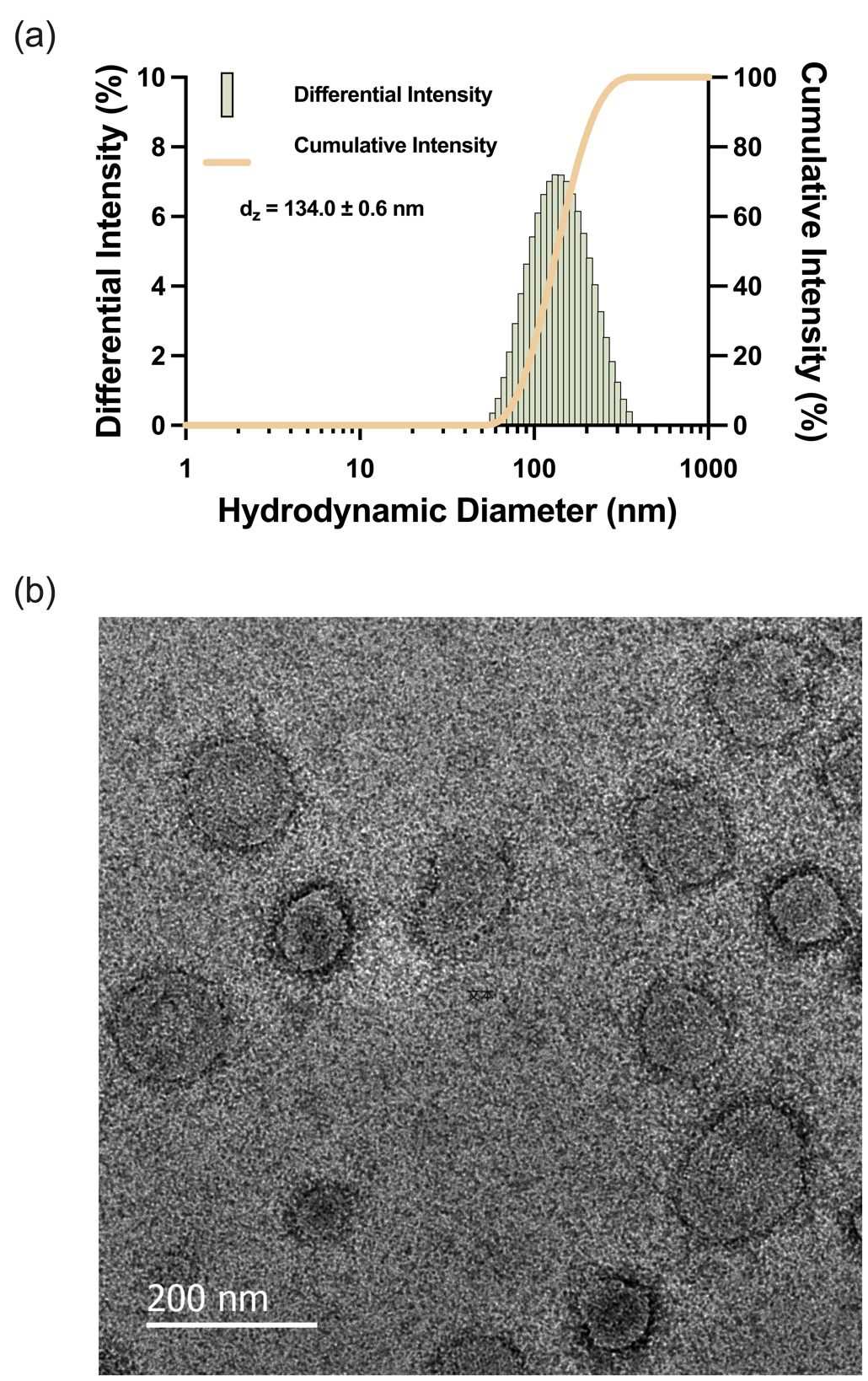


**Fig. S1.** (a) The intensity-weighted size distribution of the optimized FIN nanoparticle. (b) TEM figure of optimized FIN nanoparticle at 19,500 magnification, scale bar = 200 nm.


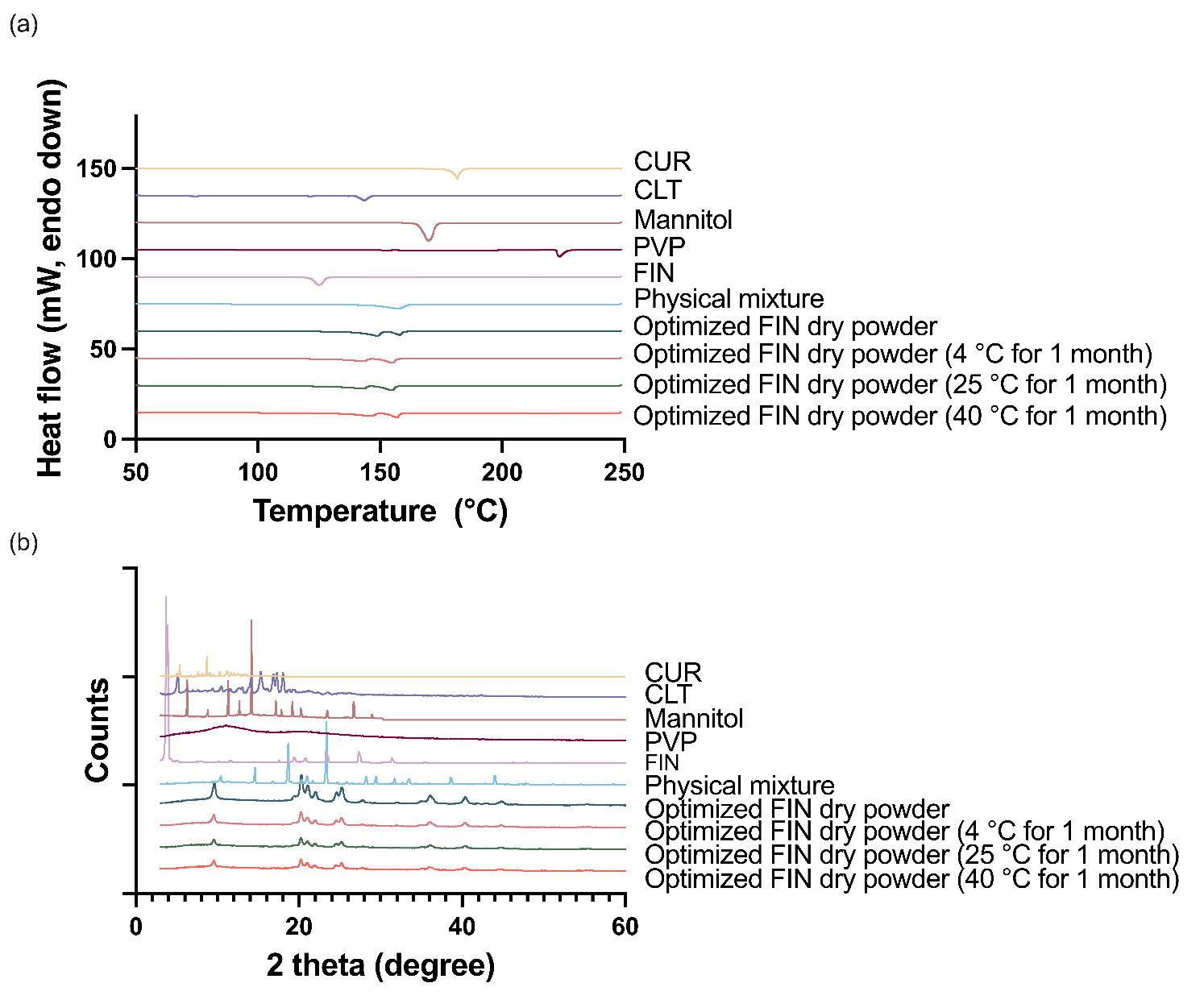

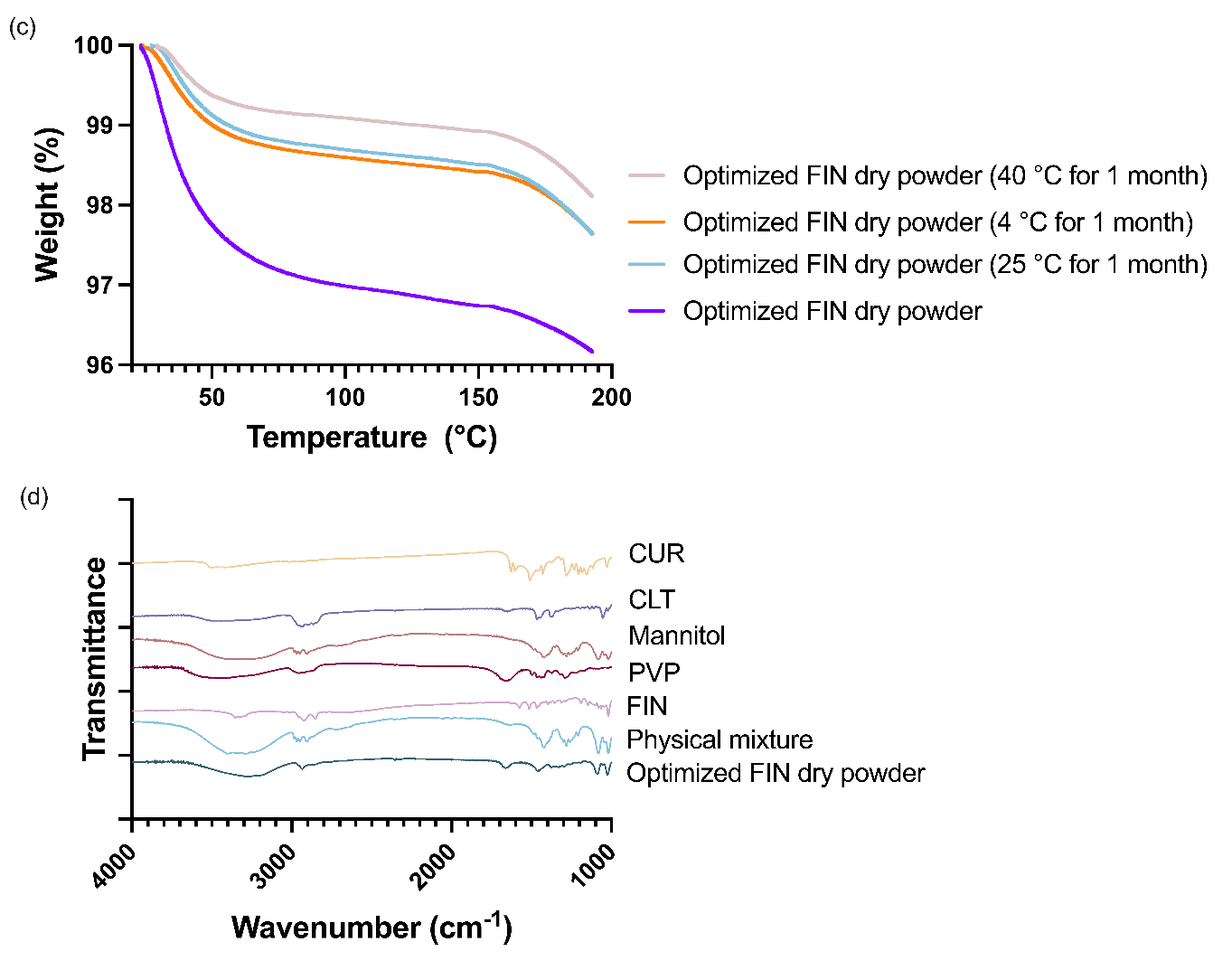


**Fig. S2.** (a) DSC profiles, (b) PXRD patterns, (c) TGA profiles, and (d) FTIR profiles of the raw materials, physical mixture, and the optimized FIN powder.


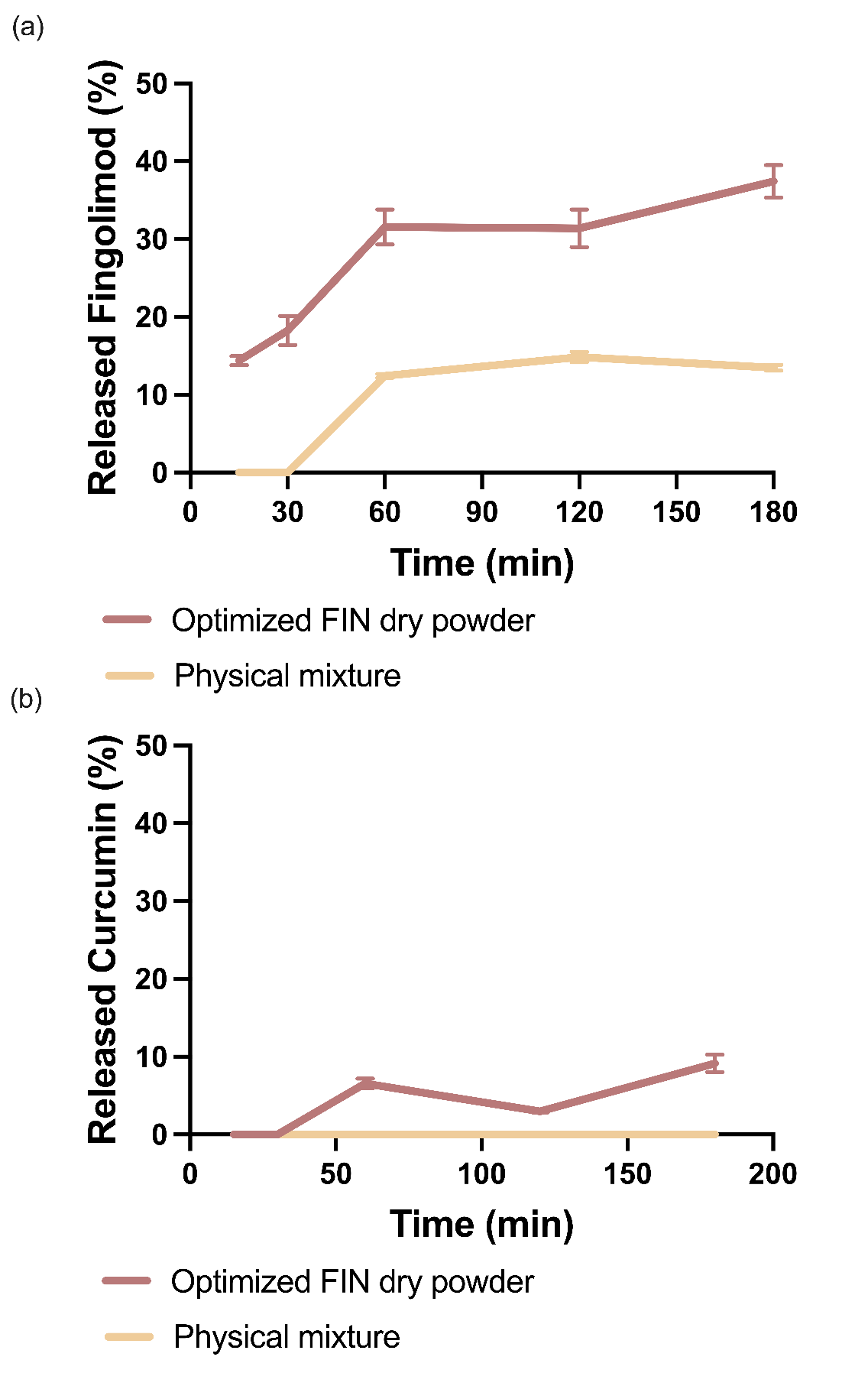


**Fig. S3.** The *in vitro* drug release of (a) FIN and (b) CUR from the optimized FIN dry powder and its physical mixture in an 80:20 v/v solution of simulated nasal fluid (SNF) and ethanol (EtOH).


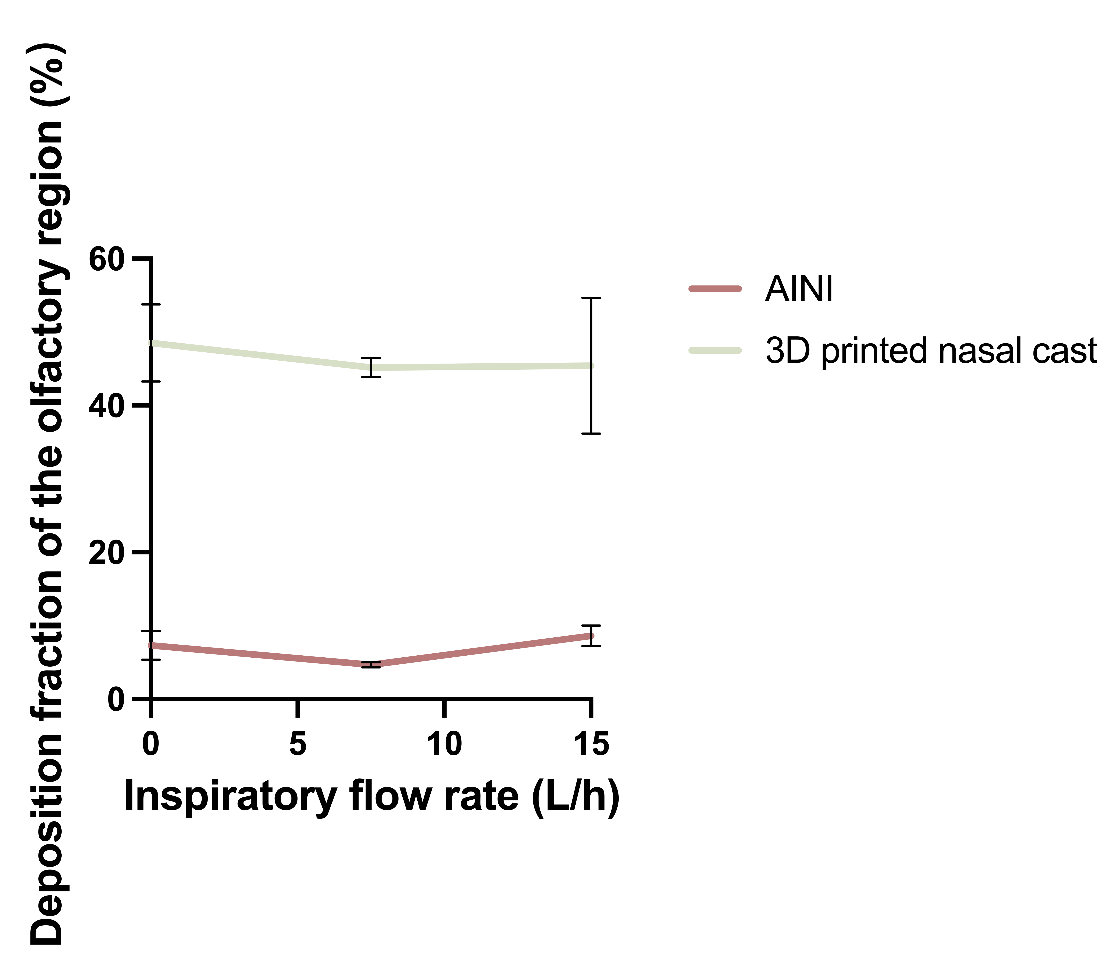


**Fig. S4.** The deposition fraction of the olfactory region at inspiratory flow rates (0, 7.5 and 15 L/min) using 3D-printed nasal cast and AINI nasal model.
